# Eggshell-Activated Carbon from Water Hyacinths for Heavy Metal Removal from Wastewater: Isotherm and Kinetic Studies

**DOI:** 10.3390/jox16040126

**Published:** 2026-07-08

**Authors:** Claire Atumanye, Simon Bbumba, Hakimu Nsubuga, Ivan Kiganda, Timothy Omara, Justus Kwetegyeka

**Affiliations:** 1 Department of Chemistry, Faculty of Science, Kyambogo University, Kyambogo P.O. Box 1, Uganda; claireatumanye01@gmail.com; 2 Department of Chemistry, College of Natural Sciences, Makerere University, Kampala P.O. Box 7062, Uganda; sbbumba762@gmail.com; 3 Department of Science, Faculty of Science and Computing, Ndejje University, Kampala P.O. Box 7088, Uganda; 4 Department of Chemistry, Faculty of Science, Muni University, Arua P.O. Box 725, Uganda; h.nsubuga@muni.ac.ug

**Keywords:** adsorption, *Eichhornia crassipes*, kinetics, optimization

## Abstract

Heavy metals (HMs) such as copper (Cu), lead (Pb), cadmium (Cd), chromium (Cr) and zinc (Zn) from industrial activities are discharged into nearby water resources after treatment. In the present study, the potential of utilizing chemically activated carbon derived from water hyacinths as a sustainable and low-cost adsorbent for heavy metal removal from industrial wastewater from the Nakawa industrial area, Uganda was investigated. The measured physicochemical parameters of wastewater (temperature, pH, electrical conductivity, total dissolved solids, turbidity, dissolved oxygen, chlorides and total hardness) varied significantly among the three sampled sites (*p* < 0.05), except for pH. Similarly, the concentration of the HMs in the samples (0.54 ± 0.04 mg L^−1^ for Cr to 93.54 ± 0.07 mg L^−1^ for Pb) varied significantly between sites (*p* < 0.05), exceeding the maximum permissible limits of Cd, Pb, Cr, Cu and Zn specified in the National Environment Standards for Discharge of Effluent into Water or Land. The water hyacinth biomass was activated using eggshell powder and phosphoric acid, followed by thermal treatment. Characterization using Fourier-transform infrared spectroscopy and scanning electron microscopy confirmed that there was improvement in its surface functionality and porosity post activation. Batch adsorption experiments indicated that optimal removal of the HMs was achieved at pH 4–5, contact time of 90 min, and 1.0 g of adsorbent. Maximum adsorption capacities of Pb, Cd, Cu, Cr and Zn were in the range of 1.04–8.36 mg g^−1^. Under the optimized conditions, the eggshell-activated carbon derived from water hyacinths had removal efficiencies of 91.2 ± 9.1% (range: 71.3–100%). Adsorption occurred through both monolayer and multilayer coverage, as indicated by the experimental data which fitted well to the Freundlich isotherm (Cd^2+^, Pb^2+^, Zn^2+^ and Cu^2+^ ions) and Langmuir isotherm model (Cr^3+^ ions). These results support the potential of water hyacinth-derived activated carbon as an ecofriendly alternative for treating low concentrations of these HMs in industrial wastewater.

## 1. Introduction

Heavy metals (HMs), toxic metals or potentially toxic elements as they are interchangeably called refer to a group of metals and metalloids that have relatively high densities and are toxic even at parts per billion concentrations [1]. Most HMs occur naturally in the earth’s crust, but their concentrations can get enriched in environmental matrices due to various anthropogenic activities [2,3]. In urban and industrialized regions, wastewater (WW) streams serve as carriers of HMs such as cadmium (Cd), copper (Cu), lead (Pb), chromium (Cr), and zinc (Zn) [4,5,6].

In addition to being persistent and non-biodegradable environmental contaminants, HMs can bioaccumulate in the food chain, resulting in amplified toxic ecological and human health effects [7,8]. The world’s worst record of HMs poisoning was the so-called Minamata disease, a severe neurological syndrome induced by methylmercury poisoning from consumption of contaminated seafood following industrial release of waste into Japan’s Minamata Bay around 1950–1960. This unforgettable incident caused at least 500 deaths, severe disabilities, congenital defects and adverse acute-toxicity symptoms like ataxia, numbness, and paralysis [9].

In developing countries of Africa and Asia, heavy metal pollution of environmental matrices is more widespread due to weak regulatory infrastructure [10,11]. In Uganda (Eastern Africa), where the industrial business park model has been adopted, studies have reported deterioration of water quality due to WW discharges into nearby streams and the surrounding Lake Victoria [12,13]. The Nakawa industrial area in Kampala city is a hotspot for industrial effluents rich in HMs. It is known for its mix of small, medium, and large industries. However, most conventional WW treatment methods employed in these industries, such as chemical precipitation, ion exchange and membrane filtration, are either expensive, technically demanding, or inefficient at low contaminant concentrations [14].

In response to the limited resources and capacity of WW treatment plants, recent research has focused on biosorption using low-cost and locally available materials. Among these, the water hyacinth (*Eichhornia crassipes*), an invasive species, has been seen as a dual opportunity to turn a noxious weed into a useful resource. This weed is beneficial due to its high lignocellulosic content and surface functionality [15], which makes it a promising precursor for activated carbon production [16].

Previous studies have demonstrated the suitability of water hyacinth for the phytoremediation and biosorption of HMs (Cd, Cr, As, Pb, Zn, Mn and Cu), with removal efficiencies ranging from 59 to 92% [16,17,18,19,20]. The current study investigated the use of chemically activated carbon derived from water hyacinth biomass to adsorb HMs from industrial WW. Activation was carried out using phosphoric acid and eggshell powder, both of which are inexpensive and environmentally benign.

## 2. Methods

### 2.1. Water Hyacinth Collection and Preparation

Whole water hyacinth plants were collected from the shores of Lake Victoria in Jinja, Uganda, washed onsite to remove mud, and packed in plastic bags. The water hyacinths were cut into small pieces (2–3 cm), oven-dried at 120 °C for 12 h, cooled and then soaked in 0.25 M ethylenediaminetetraacetic acid (at pH of 10) for 24 h. They were washed with distilled water several times until the rinse reached pH 7. This was followed by oven drying at 110 °C for 12 h. The plant material was left to cool and then ground to obtain a fine powder. The powder was sieved through 300 µm and 425 µm mesh sieves [21].

### 2.2. Preparation of the Activating Agent from Chicken Eggshells

Chicken eggshells (1 kg) were obtained from a local rolex vendor along Nakawa-Ntinda Road, Kampala. Preparation of the eggshell activating agent followed the procedure by Zhang et al. [22]. Briefly, the eggshells were washed thoroughly under running tap water to remove the membranes and organic residues. The shells were rinsed with distilled water, spread on aluminum foil, and oven-dried at 120 °C overnight. They were cooled to room temperature and ground to obtain a fine powder, which was sieved through 300 µm and 425 µm mesh sieves.

### 2.3. Sampling and Analysis of WW

Samples were collected at the exit tubes of three different industries which included: a pharmaceutical manufacturing industry (Site 1), a paint manufacturing factory (Site 2), and a battery manufacturing company (Site 3). The samples, 5 L each, were collected in labeled polyethylene bottles in March 2024 at 9:00 am, 2:00 pm and 6:00 pm from each of the sites to capture variability in discharge patterns. Non-conservable physicochemical parameters (temperature, pH, electrical conductivity, total dissolved solids), turbidity, dissolved oxygen, chlorides and total hardness of the WW samples were determined following standard methods [23].

The samples were digested using aqua regia as described by Turek et al. [24]. They were filtered through 0.42 µm filter papers, and then diluted to the 100 mL mark in volumetric flasks. Heavy metal analysis proceeded on a Shimadzu Electro Thermal Graphite Furnace Atomic Absorption Spectrometer (GF-AAS, Model AA-6300, Shimadzu Corporation, Tokyo, Japan). The wavelengths used were 283.3, 228.8, 324.7, 213.9 and 357.9 nm for Pb, Cd, Cu, Zn and Cr, respectively. Metal concentrations were determined using calibration curves created from metal salts of the HMs (Table S1). All analyses were performed in triplicate, and the corresponding blank concentrations were subtracted whenever appropriate.

### 2.4. Activation, Carbonization and Characterization of Water Hyacinth-Based Biochar

Biochar activation followed the procedure by Zięzio et al. [25] with slight modifications. Briefly, 50 g of eggshell powder and 50 g of water hyacinth powder were mixed, and to it was added 50% phosphoric acid solution while stirring continuously with a glass rod until a homogeneous slurry was formed. The mixture was then stirred for an additional 30 min to ensure that it was well impregnated with the acid solution. Following this, the beaker was covered with parafilm and the slurry left to sit at room temperature for 24 h. Carbonization of the impregnated mixture was performed as described by Danish et al. [26].

The adsorbent was prepared by treatment of water hyacinth powder (WH) with both eggshell powder (EP) and phosphoric acid (PA) to form a double-activated adsorbent (EP–WH–PA). Two control adsorbents (comprising WH and EP in a ratio of 1:1, i.e., EP–WH, and WH after carbonization, labeled WHB) were prepared in the same way, but without phosphoric acid treatment.

Surface morphology and elemental composition of the prepared water hyacinth-derived activated adsorbents were analyzed using a Zeiss Sigma 300 VP scanning electron microscope coupled with an energy-dispersive X-ray spectrometer (SEM-EDX) (Carl Zeiss GmbH, Oberkochen, Germany). Adsorbent powders were mounted on a carbon tape and imaged at an accelerating voltage of 5–20 kV.

Fourier-transform infrared (FTIR) spectroscopy of the adsorbents was performed on an Alpha II FTIR spectrometer (Bruker Optik GmbH, Ettlingen, Germany) in the Attenuated Total Reflectance mode. Spectra were recorded for both the pristine and activated adsorbents in the range of 4000–400 cm^−1^ at a spectral resolution of 4 scans per second.

### 2.5. Batch Adsorption Studies

The adsorption parameters studied included the nature of the adsorbent, particle size, pH, contact time, mass of the adsorbent, and particle size (Text S1). FTIR spectroscopy was done on the dried and ground adsorbents after adsorption experiments.

### 2.6. Adsorption Kinetic and Isotherm Models

Adsorption isotherms illustrate the relationship between how much of a substance is adsorbed onto an adsorbent and the concentration of that substance. These isotherms are equilibrium equations, valid after the adsorbate and adsorbent have interacted long enough at a stable temperature. The parameters of these equilibrium models give clues about the sorption mechanism, sorbent surface properties, and affinity. Non-linear and linear kinetic models (pseudo-first order, and pseudo-second order) were explored (Table 1). The adsorption isotherm models used in this study included Langmuir and the Freundlich models, as applied in previous studies [19,20,27].

### 2.7. Statistical Analysis

Quantitative data were assessed for normality using the Shapiro–Wilk test. The HM concentrations and other selected physicochemical parameters of the WW samples were compared with the national and international permissible limits. One-Way Analysis of Variance (ANOVA) was used to compare the mean physicochemical parameters and concentrations of the HMs in the WW samples to determine whether they differed significantly among sites. All statistical evaluations and linear fitting of the isotherm and kinetic models were performed at 95% confidence interval in Origin Pro 2026 (OriginLab Corporation, Northampton, MA, USA).

## 3. Results and Discussion

### 3.1. Physicochemical Parameters of the WW Samples

The measured physicochemical parameters (Figures S1 and S2) varied significantly by sampling site (*p* < 0.05), except for the pH. The highest mean temperature (33.7 ± 1.0 °C) was recorded in samples from Site 1 while the lowest was in samples from Site 2 (29.3 ± 0.6 °C). All the mean temperatures recorded did not exceed the permissible limits stipulated by the Uganda National Environment Management Authority (20 °C to 35 °C) [28] for WW discharge. The high temperature of WW samples in the present study may be attributed to the industrial use of water (a liquid with high specific heat capacity) to cool down equipment and manufacturing processes. Furthermore, some industrial chemical reactions are exothermic, meaning that they release heat which can raise the temperature of the water used in production processes. For example, the production of active pharmaceutical ingredients can involve exothermic reactions [29]. Hot water is also used for cleaning and sterilizing equipment to meet industrial hygiene standards [30]. Industrial operations such as distillation, crystallization, and drying are common in the pharmaceutical industry. These processes are energy intensive, and energy is often transferred to the WW [31].

For pH, all the values obtained were slightly acidic with no significant variations observed (*p* > 0.05). Nonetheless, the pH values were still within the national guidelines (6.0–8.0) [28], the WHO limits (6.5–8.5) and EU pH limits (6.5–9.5) for environmental discharge. The reason for the slight acidity could be due to organic matter from the WW components. When bacteria break down organic matter, they generate carbon dioxide gas, which lowers pH levels [32]. Earlier studies in the same study area recorded comparable pH values (6.0–6.8) [33,34], which were also close to the 6.2–7.9 for WW from Kampala Industrial and Business Park (KIBP) [12]. Previous studies linked acidic to slightly alkaline pH values of effluents from factories dealing in beverages to the nature of the raw materials such enzymes, lactic acid, benzoic acid and yeasts that are commonly used in such industries [34,35,36].

Samples from Site 3 had the highest mean EC value (826.7 ± 12.2 µS cm^−1^), followed by Sites 2 and 1 with EC of 324.1 ± 7.5 µS cm^−1^ and 223.7 ± 0.8 µS cm^−1^, respectively. The measured TDS (154.4 ± 9.8–670.3 ± 2.5 mg L^−1^) followed the same trend, with mean values varying significantly (*p* < 0.05) across sites. None of these EC and TDS values were higher than the recommended limits of 1500 µS cm^−1^ and 1000 mg L^−1^ by the WHO [37]. The high EC and TDS in Site 3 samples could be attributed to chemical additives and electrolytes such as sulfuric acid in lead-acid batteries and potassium hydroxide in alkaline batteries. The presence of HMs and other ions such as sulfates and chlorides used in industrial processes can contribute to the high EC of wastewater [38]. Previous studies of WW and impacted stream water from industrial areas in Uganda reported EC and TDS values of 80.5–45,000 µS cm^−1^ and 47.0–370.7 mg L^−1^ [12,33,39], which are higher than in the present study.

For turbidity, samples from Site 2 had the highest mean value (764.7 ± 0.8 NTU) while Site 3 had the least measured value (298.5 ± 6.9 NTU). Such turbidity values are linked to high levels of suspended matter that come from pharmaceutical, paint, and battery WW components during the manufacturing process. Earlier studies [12,33,39] recorded turbidity values of 10.7–715.9 NTU in effluents and the associated stream water in Uganda, which are comparable to those obtained in the present study. In WW, high turbidity indicates the presence of particles that can shelter harmful organisms from disinfection processes, creating difficulties with compliance with discharge limits [40].

Dissolved oxygen varied between 6.5 ± 0.7 mg L^−1^ and 9.3 ± 0.6 mg L^−1^, which were below the required EU limits for dissolved oxygen (10–12 mg L^−1^). The low dissolved oxygen values could be attributed to the high temperature of the WW samples [41]. In addition, the metabolic activities of aerobic bacteria increase with increase in temperature. Thus, at higher WW temperatures, they use up oxygen more quickly. As a result, not only might the amount of oxygen in the water decrease, but it may also deplete faster [42].

The mean concentration of chlorides differed significantly (*p* < 0.05) across the three sampling sites, and none of the mean values obtained were compliant with the standard regulatory concentration for domestic water for chlorides (250 mg L^−1^) set by both the WHO and the EU. The highest mean chloride concentration was recorded in Site 1 (1592.1 ± 19.8 mg L^−1^), followed by Site 2, which had a concentration of 1608.7 ± 8.3 mg L^−1^, while Site 3 had the least (427.8 ± 8.5 mg L^−1^). These concentrations were one to two orders of magnitude higher than the 11.68–31.08 mg L^−1^ previously reported for effluent-impacted stream water in the Nakawa-Ntinda industrial area [33]. High concentrations of chlorides in pharmaceutical industry wastewater are mainly due to the use of chlorinated compounds, for example, hydrochloric acid, methylene chloride and sodium chloride in manufacturing processes [43].

Considering total hardness, the values obtained varied from 218.0 ± 4.9 mg L^−1^ in samples from Site 2 to 353.2 ± 6.3 mg L^−1^ in samples from Site 1. These mean total hardness values exceeded the regulatory limit of 200 mg L^−1^ set by the WHO. The high total hardness recorded in samples across the three sites could be due to the extensive use of chemicals, solvents, and minerals containing Ca and Mg ions in the manufacturing processes [44]. Considered together, the measured physicochemical parameters of the WW were in the range reported by previous authors in other parts of the world, except dissolved oxygen (Table 2).

### 3.2. Concentration of HMs in WW

The concentration of the HMs in the WW samples (0.54 ± 0.04 mg L^−1^ for Cr^3+^ to 93.54 ± 0.07 mg L^−1^ for Pb^2+^) varied significantly between sites (*p* < 0.05; Table 3). There was site-specific dominance observed for some of the HMs. For example, Cd^2+^ and Pb^2+^ ions were quantified at the highest concentrations in samples from Site 3 whereas Cr^3+^, Zn^2+^ and Cu^2+^ ions had the highest concentrations in samples from Site 2, which could reflect inputs from battery manufacturing and paint-related industrial activities. Most of the measured HM concentrations were several folds higher than the maximum permissible limits of 0.01, 0.1, 0.5, 0.5 and 2.0 mg L^−1^ for Cd, Pb, Cr, Cu and Zn as specified in schedule 3 of the National Environment (Standards for Discharge of Effluent into Water or Land) [48]. This indicates very high HM contamination, especially for Cd and Pb.

Compared to previous studies from industrial areas of Uganda, the concentrations of HMs obtained in the present study are much higher. For example, Walakira and Okot-Okumu [33] quantified Pb (0.039–0.256 mg L^−1^) and Cu (0.015–0.52 mg L^−1^) in stream water samples from the same study area whereas Cd was not detected. In the Nairobi industrial area (Kenya), WW samples were reported to contain Cd (0.00008–0.00017 mg L^−1^), Cr (0.0007–0.0507 mg L^−1^) and Pb (0.0004–0.0295 mg L^−1^) [45]. Another study in Michigan (USA), quantified Cr (0.00033–0.00042 mg L^−1^) and (0.00346 mg L^−1^) while Cd and Cu were not detected [6]. Similar lower HM concentrations (0.008–0.038 mg L^−1^, 0.40–0.254 mg L^−1^, 0.046–0.096 mg L^−1^ and 0.043–1.355 mg L^−1^) were reported for Cd, Cr, Pb and Zn in WW from Hayatabad, Pakistan [46].

### 3.3. Characterization of Adsorbents

**Figure 1:**
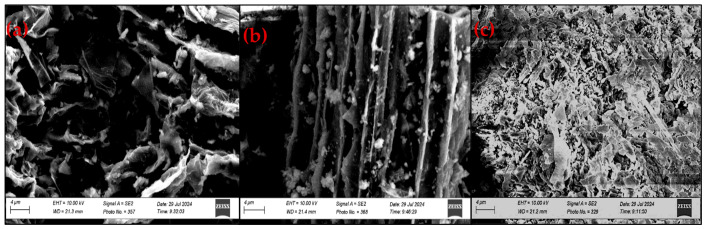
Scanning electron micrographs of adsorbents: (**a**) unactivated water hyacinth biochar (WHB), (**b**) eggshell-treated activated water hyacinth biochar (EP–WH), and (**c**) double activated water hyacinth biochar (EP–WH–PA).

Surface morphology of the adsorbents was highly porous, as evidenced by their uneven and rough surface. Sponge-like pore structures were formed on the surface of EP–WH during activation and calcification (Figure 1). Eggshell powder contains calcium carbonate particles, and because of their larger atomic number, these particles tend to appear as bright spots [49].

In EP–WH–PA, the adsorbent surface developed pore patterns resembling honeycombs after activation with phosphoric acid. The evaporation of acid during carbonization created pores of various sizes and shapes in areas previously occupied by the acid. Phosphoric acid-activated biochar exhibited enhanced porosity and unique pore structures, indicating an increased surface area and pore volume [50,51]. The first step in the treatment of the adsorbent with phosphoric acid involved the depolymerization of cellulose, which was followed by dehydration, formation of aromatic rings, and, finally, the elimination of phosphate clusters, leaving a porous structure [52].

**Figure 2:**
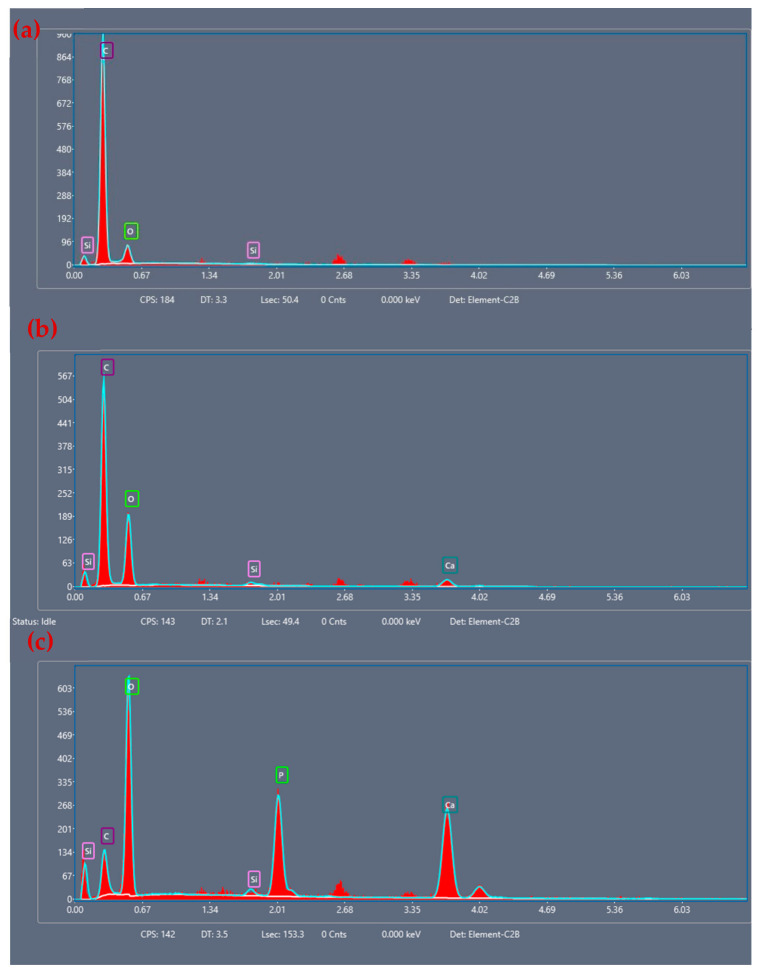
Energy dispersive X-ray spectroscopy spectrum of (**a**) unactivated water hyacinth biochar (WHB), (**b**) eggshell-treated activated water hyacinth biochar (EP–WH), and (**c**) double activated water hyacinth biochar (EP–WH–PA).

The energy-dispersive X-ray spectroscopy spectra and elemental composition of the prepared water hyacinth-based adsorbents are shown in Figure 2 and Table S2. Elements (C, O, Si, P, and Ca) were present in the activated carbon adsorbents. This signified successful incorporation of both calcium and phosphorus groups into the adsorbent [53]. The highest percentage of carbon was found in the unactivated adsorbent. This can be attributed to incorporating calcium carbonate from eggshell powder and phosphate groups from phosphoric acid treatment, which introduced significant amounts of non-carbon elements, reducing the relative carbon content [54].

The FTIR spectra also showed the presence of additional functional groups in the activated biochars (Figure S3). In the spectrum of the unactivated water hyacinth biochar (WHB), the broad peak at around 3200–3600 cm^−1^ can be attributed to the OH stretching vibration mode of the hydroxyl functional groups, arising from the presence of cellulose and lignin [15]. There was no peak around 2900 cm^−1^, which is often observed in the FTIR spectrum of raw water hyacinth. This indicates the decomposition of aliphatic hydrocarbons due to pyrolysis. The peak at 1423 cm^−1^ in the FTIR spectrum of water hyacinth biochar is indicative of the stretching vibrations of aromatic carbon-carbon (C=C) bonds, which are commonly found in biochars due to the formation of stable aromatic structures during pyrolysis [55]. The peak at 1038 cm^−1^ could be attributed to phenolic C-O and O-H stretching, indicating the presence of alcohols, ethers, or esters, common in cellulose and hemicelluloses. It could also correspond to the Si-O-Si stretching vibrations due to the presence of silica-derived compounds in the inorganic content within the biochar matrix. The peaks at 872 cm^−1^ and 566 cm^−1^ in the FTIR spectrum of the water hyacinth biochar are typically attributed to Si-O-Si bending vibrations, characteristic of silicate minerals derived from inorganic silica in plant tissues [56]. At 713 cm^−1^, the peak is indicative of the out-of-plane bending vibrations of C-H bonds in aromatic rings common in biochars due to the thermal decomposition of lignocellulosic materials.

In the eggshell-treated water hyacinth biochar (EP–WH), the broad peak around 3200–3600 cm^−1^ was reduced, indicating a suppression of the OH stretching. This results from dehydration and loss of hydroxyl groups due to pyrolysis [57]. All the peaks were shifted, except the one at 872 cm^−1^ (the Si-O-Si stretch). However, the intensity of this peak increased due to the addition of silicate from eggshell powder [58]. The shifting in the C=C stretching of the peak at 1413 cm^−1^ to 1415 cm^−1^ indicated an increase in aromatic C=C stretching of the Ca-WH bond. Furthermore, the peaks around 1000–1300 cm^−1^ shifted, indicating changes in oxygenated functional groups due to thermal degradation and interactions with eggshell powder. The peak at 713 cm^−1^ in the unactivated water hyacinth shifted to 709 cm^−1^ in the Ca-WH and also increased in intensity, due to an increase in the C-H bonds of the aromatic rings. The peak at 566 cm^−1^ also experienced a minor shift to 565 cm^−1^, and new peaks were created around the same position, potentially corresponding to new Ca-O bonds. These suggested the successful incorporation of Ca from eggshell powder into the biochar matrix.

For the phosphoric acid activated-calcium doped water hyacinth biochar (EP–WH–PA), there was a disappearance of the broad O-H stretching around 3200–3600 cm^−1^, indicating complete dehydration. New peaks around 937–1143 cm^−1^ were created, indicating P-O stretching vibrations from phosphates introduced during activation with phosphoric acid. The peak at 872 cm^−1^ disappeared due to interaction with phosphoric acid, with the Metal-O bond established at this position. There was an increase in the intensity of the peak at 565 cm^−1^ and the emergence of new peaks below this wavelength to around 449 cm^−1^. These observed peaks are associated with Ca-O stretching and P-O bending vibrations. In the Ca-O context, the peaks indicate the presence of calcium carbonate from the eggshell powder within the biochar structure [59]. The P-O bending vibrations in this region indicate the successful activation of the biochar with phosphoric acid, integrating phosphate groups into the structure [60]. The FTIR results revealed carbon-containing functional groups, which were in agreement with the energy-dispersive X-ray spectroscopy results.

### 3.4. Adsorption Efficiency and Adsorption Capacity of Activated Water Hyacinth Powder

The adsorption efficiency and adsorption capacity of activated water hyacinth varied with the initial HM concentration and the adsorbent. Lower adsorption efficiencies were observed at higher HM concentrations (Table S3). This may be attributed to progressive saturation of the available adsorption sites at high concentrations, resulting in increased competition among metal ions for active binding sites on the adsorbent surface [61]. Adsorption capacity increased with increasing metal concentration, plausibly due to the higher concentration gradient between the solution and adsorbent surface at higher concentrations, which provides a greater driving force for mass transfer and adsorption [62].

For a given HM concentration, adsorption efficiencies obtained in aqueous solutions were higher than those observed in the WW samples. The reduced adsorption efficiency in WW stems from the presence of competing ions and other dissolved constituents that interfere with the adsorption of target HMs onto the active sites of the adsorbent. In aqueous solutions, a single metal species was present, thereby minimizing competitive adsorption effects [63].

#### 3.4.1. Effect of Adsorbent Type

The EP–WH–PA achieved the highest adsorption efficiency for the HMs, followed by EP–WH and then WHB (Table S3). This indicates that combined activation of WHB with eggshell powder and phosphoric acid enhanced the adsorption performance of the adsorbent. The improved adsorption could thus be attributed to the introduction of additional functional groups and the enhancement of surface properties resulting from chemical modification. Eggshell treatment introduces calcium-containing compounds onto the adsorbent surface, while phosphoric acid activation promotes the development of a rougher and more porous surface morphology, as observed in the SEM images, thereby providing more accessible adsorption sites for the metal ions. In addition, the oxygen-containing functional groups identified by FTIR can facilitate electrostatic attraction, surface complexation, and ion exchange with the metal ions [64]. The EP–WH–PA adsorbent was thus selected for subsequent optimization studies.

The FTIR spectra of the EP–WH–PA adsorbent before and after adsorption of the selected metal ions were compared to elucidate the interaction between surface functional groups and the metal ions. Loading of the activated adsorbent with Cu^2+^ resulted in the disappearance of the peak at 1415 cm^−1^ and a reduction in the intensity of peaks around 565 cm^−1^ and 480 cm^−1^, indicating the involvement of phosphate groups and calcium sites in Cu^2+^ binding. Similar shifts and reductions in peak intensities were observed after adsorption of the other metal ions, demonstrating interactions between the functional groups of the biochar and the adsorbed metal ions (Figure S4). These spectral changes confirmed the successful adsorption of the HM ions onto the eggshell-activated carbon derived from water hyacinth.

#### 3.4.2. Effect of Adsorbent Particle Size

Adsorption efficiency increased with decreasing particle size (Table S4). The highest adsorption efficiencies were obtained using the 300 µm adsorbent particles, which could be inextricably attributed to the increased surface area in adsorbents with smaller particle sizes. It is also known that reduction in adsorbent particle size decreases intraparticle diffusion resistance, thereby enhancing adsorption kinetics [65].

Complete adsorption (100%) was achieved for low-concentrations using the 300 µm adsorbents, demonstrating the strong adsorption potential of activated water hyacinth for dilute HM solutions. Adsorbent particles smaller than 300 µm were thus used in the subsequent adsorption experiments.

#### 3.4.3. Effect of Solution pH

Solution pH is one of the most important factors influencing HM adsorption because it affects both the surface charge of the adsorbent and the speciation of HM ions in solution. The degree of protonation or deprotonation of functional groups such as hydroxyl and carboxyl groups on the adsorbent surface strongly influences adsorption behavior. The adsorption efficiency of Cd^2+^ ions increased as the pH increased from 3 to 5 (Table S5). As the pH increased, proton competition decreased, and the surface functional groups became progressively deprotonated, thereby enhancing electrostatic attraction and complexation between Cd^2+^ ions and the adsorbent surface. Maximum adsorption was observed at pH 5, above which adsorption efficiency increased only slightly, suggesting gradual saturation of adsorption sites.

The adsorption of Pb^2+^, Zn^2+^, Cr^3+^, and Cu^2+^ ions followed similar trends. Maximum adsorption of Zn^2+^ and Cd^2+^ occurred at pH 5, whereas Pb^2+^, Cu^2+^, and Cr^3+^ exhibited optimum adsorption at pH 4. The decrease in adsorption efficiency observed at higher pH values for Pb^2+^, Cu^2+^, and Cr^3+^ may be attributed to the formation of insoluble metal hydroxides such as Pb(OH)_2_, Cu(OH)_2_, and Cr(OH)_3_. Under such conditions, HM removal proceeds primarily through precipitation rather than adsorption [66].

#### 3.4.4. Effect of Contact Time

The adsorption of Cd^2+^ ions increased with increasing contact time (Table S6). Rapid adsorption occurred during the initial stages of the process due to the abundance of vacant adsorption sites on the adsorbent surface. Thereafter, the adsorption rate gradually decreased as the available active sites became occupied and equilibrium was approached. For the low-concentration Cd^2+^ solution (7.54 mg L^−1^), adsorption equilibrium was achieved within 10 min, after which adsorption efficiency remained constant at 96.8%. The rapid attainment of equilibrium at low concentration may be attributed to reduced competition among metal ions for available adsorption sites. The adsorption process, therefore, involved an initial rapid surface-adsorption stage followed by a slower diffusion-controlled stage associated with intraparticle diffusion and the progressive occupation of adsorption sites.

#### 3.4.5. Effect of Adsorbent Dosage

Adsorbent dosage is an important operational parameter because it affects the number of available adsorption sites and thus influences the adsorption efficiency. In the present study, adsorption efficiency increased with increasing adsorbent dosage (Table S7). This increase follows from the higher number of active sites available for metal ion binding at higher adsorbent masses.

For Cd^2+^ solutions of 7.54 mg L^−1^, complete adsorption was achieved using 0.75 g of adsorbent, indicating sufficient availability of adsorption sites for total removal of metal ions at low concentrations. Optimum adsorption for Cd^2+^ ions at concentrations of 40.18 mg L^−1^ and 77.13 mg L^−1^ was obtained using 0.75 g and 1.0 g of adsorbent, respectively. This trend was also observed for the other HMs. The optimum adsorption for lead was at 1.25 g while that of Zn^2+^, Cu^2+^ and Cr^3+^ ions was 1.0 g.

The increase in adsorption efficiency with adsorbent dosage may also be associated with increased surface area and enhanced availability of functional groups capable of interacting with metal ions through electrostatic attraction, ion exchange, and surface-complexation mechanisms [67].

### 3.5. Kinetic Studies

The kinetics of HM adsorption onto activated water hyacinth powder were evaluated using pseudo-first order and pseudo-second order kinetic models (Table 4; Figures S5–S9). The pseudo-second order kinetic model exhibited a higher correlation coefficient (R^2^ = 0.9813) compared to the pseudo-first order model (R^2^ = 0.9652) for Cd^2+^ adsorption. This suggests that the adsorption process was more accurately described by the pseudo-second order model.

The adsorptions of Zn^2+^ and Cr^3+^ ions were also better represented by the pseudo-second order model, indicating that adsorption kinetics were influenced by the availability of adsorption sites and interactions between the adsorbent surface and metal ions [68]. In contrast, Pb^2+^ and Cu^2+^ adsorption showed better agreement with the pseudo-first order kinetic model, suggesting that diffusion-controlled processes and physical adsorption mechanisms may have contributed significantly to their adsorption behavior [69]. Previous studies reported that the adsorption of Cd^2+^ and Mn^2+^ ions by WH biochar had a high degree of fitting with the pseudo-second order model [18,19], which is the same as observed in the current study.

These results indicate that adsorption kinetics varied depending on the specific HM ion and involved multiple mechanisms (surface adsorption, pore diffusion, electrostatic interactions, and ion exchange). Overall, the adsorption of HMs onto activated WH powder demonstrated favorable adsorption behavior and efficient uptake kinetics, confirming the potential of the prepared adsorbent for wastewater treatment applications.

### 3.6. Adsorption Equilibrium Isotherms

Adsorption isotherms describe the interaction between adsorbate molecules and the adsorbent surface and provide important information regarding adsorption mechanisms, surface properties, and adsorption capacity. In this study, the experimental data were fitted using both Langmuir and Freundlich adsorption isotherm models.

The Freundlich isotherm model generally provided a better description of the adsorption process compared to the Langmuir model (Table 5; Figures S10 and S11). The Freundlich model assumes heterogeneous surface adsorption and multilayer adsorption behavior, which is consistent with the complex surface characteristics of activated water hyacinth biochar. Although some Langmuir plots produced relatively high correlation coefficients (R^2^), the negative intercepts obtained for most of the HMs indicate that the assumptions underlying the Langmuir model (for example, monolayer adsorption onto a homogeneous surface with identical adsorption sites) were not fully satisfied.

The Freundlich model provided a better fit for Cd^2+^, Pb^2+^, Zn^2+^ and Cu^2+^ adsorption, whereas the Langmuir model better described the adsorption behavior of Pb^2+^ and Cr^3+^ ions. The adsorption of Cd^2+^, Pb^2+^, Zn^2+^ and Cu^2+^ ions onto the activated water hyacinth biochar thus involved multiple mechanisms: electrostatic attraction, ion exchange, surface complexation, and pore diffusion facilitated by oxygen-containing functional groups and calcium-derived active sites introduced during chemical activation [70]. The better fit of the Langmuir model for Cr^3+^ ions showed that its adsorption occurred through monolayer coverage on relatively homogeneous adsorption sites [71]. Together, the obtained results emphasized that the adsorption mechanisms are metal-dependent and that both homogeneous and heterogeneous surface interactions contributed to the adsorption process.

These results are contrary to those of Wang et al. [19] who reported that the adsorption of Cd^2+^ ions in wastewater by WH biochar had a high degree of fitting with the Langmuir model. In another study, the adsorption of Cr^6+^ ions by WH biomass mixed with iron chloride was reported to fit well with the Langmuir model [20]. Tesi et al. [18] found that Mn^2+^ ion biosorption by WH roots could best modeled by the Freundlich isotherm, which agrees with the results obtained for the adsorption of Cd^2+^, Pb^2+^, Zn^2+^ and Cu^2+^ ions in the current study.

The adsorption performance of the eggshell-activated carbon derived from water hyacinth was compared with those of recently reported water hyacinth-derived and other biochar-based adsorbents for heavy metal removal (Table 6). In most previous studies, the adsorption of the HMs fitted well with Langmuir model [19,20,21,27,71,72].

Although regeneration studies were beyond the scope of the present work, previous studies have demonstrated that HMs adsorbed onto carbonaceous and calcium-rich adsorbents can be desorbed using dilute mineral acids [66,73]. Future studies should therefore investigate the regeneration efficiency and reusability of the eggshell-activated carbon to enhance the sustainability of the proposed WW treatment based on the adsorbent.

## 4. Conclusions

The present study quantified high concentrations of HMs (0.54–93.54 mg L^−1^) in WW sampled from different sites within the Nakawa industrial area. Activated water hyacinth-based adsorbents were efficient at remediating HMs from wastewater. For low metal concentrations, adsorption efficiency as high as 100% was achieved by the adsorbents. Synergistically activated water hyacinth-based adsorbent had a higher adsorption efficiency compared to the adsorbent activated with a single activating agent and the non-activated water hyacinth adsorbent. The optimal adsorption of metal ions was at a pH range of 4–5, contact time of 90 min, and 1.0 g of adsorbent. Maximum adsorption capacities were 7.26, 10.21, 5.33,1.04 and 8.36 mg g^−1^ for Pb, Cd, Cu, Cr and Zn, respectively. Adsorption occurred through multilayer coverage, as indicated by the experimental data which fitted well to the Freundlich isotherm.

## Figures and Tables

**Table 1:** Non-linear and linear forms of the isotherm and kinetic models.

Model	Equations	
	Non-Linear	Linear
Pseudo-first order	$q_{t}=q_{e}\left(1-e^{\left({-k}_{1}t\right)}\right)$	$In\left(q_{e}-q_{t}\right)=Inq_{e}-k_{1}t$
Pseudo-second order	$q_{t}=\frac{q_{e}^{2}K_{2}t}{1+q_{e}K_{2}t}$	$\frac{t}{q_{t}}=\frac{1}{k_{2}q_{e}^{2}}+\frac{1}{q_{e}}t$
Langmuir	$q_{e}=\frac{q_{m}bC_{e}}{1+bC_{e}}$	$\frac{C_{e}}{q_{e}}=\frac{1}{{q_{m}k}_{L}}+\frac{C_{e}}{q_{m}}$
Freundlich	qe=kfCe1n	$logq_{e}=b_{F}logC_{e}+logk_{f}$

Note: q_e_ = equilibrium adsorption capacity, $k_{f}$ = Freundlich constant related to adsorption capacity, *n* = Freundlich constant related to adsorption intensity, *q_m_* = maximum monolayer adsorption capacity, k_L_ = solute absorptivity, b_F_ = heterogeneity constant, q_t_ = time adsorption constant, K_1_ = first-order rate coefficient, t = time, and K_2_ = second-order rate coefficient.

**Table 2:** Comparison of physicochemical parameters of wastewater samples from the Nakawa industrial area with previous studies.

Study Area	Temperature (°C)	pH	Turbidity (NTU)	TDS (mg L^−1^)	EC (μS cm^−1^)	DO (mg L^−1^)	Chlorides (mg L^−1^)	Total Hardness (mg L^−1^)	References
Nakawa (Uganda)	29.3–33.7	6.1–6.8	298.5–764.7	154.4–670.3	223.7–826.7	6.5–9.3	427.8–1608.7	218.0–353.2	Present study
Nairobi (Kenya)	16.8–26.1	7.3–8.8	–	–	336.7–1134.3	–	–	–	[45]
Michigan, USA	17.9–29.0	7.8–9.0	–	–	236–929	–	–	–	[6]
Hayatabad, Pakistan	–	6.4–7.3	–	560–942	1000–1800	–	–	–	[46]
Hattar, Pakistan	10.7–47.1	4.6–10.9	11.8–32.3	392–1021	702–1025.7	0.02–3.20	–	–	[47]

Note: – Means not determined, DO = dissolved oxygen, EC = electrical conductivity, TDS = total dissolved solids.

**Table 3:** Concentration of heavy metals quantified in wastewater samples.

Heavy Metal (Ions)	Site	Mean Concentration (mg L^−1^)	Permissible Limits (mg L^−1^) [48]
Cd^2+^	1	7.45 ± 0.02 ^a^	0.01
	2	40.18 ± 0.17 ^b^	
	3	77.13 ± 0.23 ^c^	
Cr^3+^	1	8.21 ± 0.12 ^a^	0.5
	2	13.07 ± 0.19 ^b^	
	3	0.54 ± 0.04 ^c^	
Pb^2+^	1	4.75 ± 0.03 ^a^	0.1
	2	72.03 ± 0.10 ^b^	
	3	93.54 ± 0.07 ^c^	
Zn^2+^	1	63.02 ± 0.03 ^a^	2.0
	2	84.62 ± 0.27 ^b^	
	3	0.91 ± 0.07 ^c^	
Cu^2+^	1	8.73 ± 0.08 ^a^	0.5
	2	54.03 ± 0.18 ^b^	
	3	0.64 ± 0.08 ^c^	

Note: The concentrations are expressed as mean ± standard deviation of triplicates. Means across the sampled sites for the same metal carrying different superscript letters are statistically different (*p* < 0.05).

**Table 4:** Kinetic parameters for the adsorption of the heavy metals.

Metal Ions	Pseudo-First Order Kinetics			Pseudo-Second Order Kinetics		
	K_1_ (min^−1^)	$q_{e}$ (mg g^−1^)	R^2^	K_2_ (g mg^−1^ min^−1^)	$q_{e}$ (mg g^−1^)	R^2^
Cd^2+^	0.1108	13.9508	0.9652	0.0026	19.6078	0.9813
Pb^2+^	0.2084	13.9508	0.9652	0.0042	20.202	08777
Zn^2+^	0.1147	8.533	0.941	0.0317	12.8041	0.9998
Cu^2+^	0.2142	9.7477	0.9883	0.0117	12.1655	0.9735
Cr^3+^	0.1177	1.6588	0.9297	0.1767	2.5543	0.9998

**Table 5:** Langmuir and Freundlich parameters for heavy metal adsorption.

Metal Ions	Langmuir Fitting			Freundlich Fitting		
	K_L_ (L mg^−1^)	$q_{m}$ (mg g^−1^)	R^2^	*n*	$K_{f}$	R^2^
Cd^2+^	0.167	15.74	0.953	2.17	2.92	0.998
Pb^2+^	0.032	25.60	0.917	1.27	0.94	0.971
Zn^2+^	0.077	18.42	0.986	1.61	1.68	0.987
Cu^2+^	0.035	30.52	0.981	1.23	1.18	0.988
Cr^3+^	0.193	15.79	0.973	2.07	2.95	0.958

**Table 6:** Comparison of adsorption performance of the eggshell-activated carbon derived from water hyacinth with previous studies.

Adsorbent	Part Used	Metal Ions	Langmuir Fitting			Freundlich Fitting			References
			K_L_ (L mg^−1^)	$q_{m}$ (mg g^−1^)	R^2^	*n*	$K_{f}$	R^2^	
Eggshell-activated carbon	Water hyacinth plant	Cd^2+^, Pb^2+^, Zn^2+^, Cu^2+^, Cr^3+^	0.032–0.193	15.74–30.52	0.917–0.986	1.23–2.17	0.94–2.92	0.958–0.998	Current study
Unactivated biochar		Cd^2+^	0.222–13.50	7.107–28.409	0.838–0.987	2.5–10.7	15.1–1835.2	0.042–0.832	[19]
Water hyacinth-iron chloride		Cr^6+^	0.4–0.6	50	0.98–0.99	-	0.10–0.17	0.91–0.96	[20]
Ground roots		Pb^2+^	0.8627	49.75	0.987	-	23.52	0.909	[21]
Phosphoric acid-activated root biomass		Pb^2+^	-	-	0.960	-	-	-	[27]
*Ficus natalensis* biochar	Fruit	Cu^2+^	0.188	161.3	0.648	3.604	105.58	0.9799	[71]
		Pb^2+^	0.160	1250	0.973	1.922	199.43	0.4668	
*Acacia erioloba*, *Combretum apiculatum* and *Adansonia digitata* biochars	Seeds	Pb^2+^	0.12–0.56	−0.67–34.7	0.49–0.93	0.26–1.06	7.0–42,707	0.42–0.95	[72]

Note: - means not reported.

## Data Availability

The original contributions presented in this study are included in the article/Supplementary Material. Further inquiries can be directed to the corresponding authors.

## Supplementary Materials

The following supporting information can be downloaded at: https://www.mdpi.com/article/10.3390/jox16040126/s1, Text S1: Batch adsorption studies; Table S1: Calibration curve parameters for the analysis of heavy metals in the samples; Figure S1: Non-conservable physicochemical parameters of the wastewater samples’ (a) temperature, (b) pH, (c) electrical conductivity, and (d) total dissolved solids. For each parameter, bars carrying different alphabetical letters are statistically different (*p* < 0.05); Figure S2: Conservable chemical parameters of the wastewater samples’ (a) turbidity, (b) dissolved oxygen, (c) chlorides, and (d) total hardness. For each parameter, bars carrying different alphabetical letters are statistically different (*p* < 0.05); Table S2: Elemental composition of the water hyacinth-based adsorbents; Table S3: Effect of adsorbent type on the adsorption of the HMs; Figure S3: FTIR spectrum of (a) unactivated water hyacinth biochar (WHB), (b) eggshell-treated activated water hyacinth biochar (EP–WH), and (c) double activated water hyacinth biochar (EP–WH–PA); Figure S4: FTIR spectra after adsorption of selected metal ions by the activated water hyacinth biochar (EP–WH–PA); Table S4: Effect of adsorbent particle size on the adsorption of HMs; Table S5: Effect of solution pH on the adsorption of HMs; Table S6: Effect of contact time on the adsorption of HMs; Table S7: Effect of adsorbent dosage on the adsorption of HMs; Figure S5: Linearized (a) pseudo-first order, and (b) pseudo-second order kinetic plots for the adsorption of Cd^2+^ ions (77.13 mg L^−1^) onto eggshell-activated carbon derived from water hyacinths; Figure S6: Linearized (a) pseudo-first order, and (b) pseudo-second order kinetic plots for the adsorption of Pb^2+^ ions (93.54 mg L^−1^) onto eggshell-activated carbon derived from water hyacinths; Figure S7: Linearized (a) pseudo-first order, and (b) pseudo-second order kinetic plots for the adsorption of Zn^2+^ ions (63.01 mg L^−1^) onto eggshell-activated carbon derived from water hyacinths; Figure S8: Linearized (a) pseudo-first order, and (b) pseudo-second order kinetic plots for the adsorption of Cu^2+^ ions (54.03 mg L^−1^) onto eggshell-activated carbon derived from water hyacinths; Figure S9: Linearized (a) pseudo-first order, and (b) pseudo-second order kinetic plots for the adsorption of Cr^3+^ ions (13.07 mg L^−1^) onto eggshell-activated carbon derived from water hyacinths; Figure S10: Linearized Langmuir isotherm plots for the adsorption of (a) Cd^2+^, (b) Pb^2+^, (c) Zn^2+^, (d) Cu^2+^, and (e) Cr^3+^ ions onto eggshell-activated carbon derived from water hyacinths; Figure S11: Linearized Freundlich isotherm plots for the adsorption of (a) Cd^2+^, (b) Pb^2+^, (c) Zn^2+^, (d) Cu^2+^, and (e) Cr^3+^ ions onto eggshell-activated carbon derived from water hyacinths.

## References

[1] Pourret O., Hursthouse A. (2019). It’s Time to Replace the Term “Heavy Metals” with “Potentially Toxic Elements” When Reporting Environmental Research. Int. J. Environ. Res. Public Health.

[2] Witkowska D., Słowik J., Chilicka K. (2021). Heavy Metals and Human Health: Possible Exposure Pathways and the Competition for Protein Binding Sites. Molecules.

[3] El Ouaty O., El M’rini A., Nachite D., Marrocchino E., Marin E., Rodella I. (2022). Assessment of the heavy metal sources and concentrations in the Nador Lagoon sediment, Northeast-Morocco. Ocean Coast. Manag..

[4] El-Sharkawy M., Alotaibi M.O., Li J., Du D., Mahmoud E. (2025). Heavy Metal Pollution in Coastal Environments: Ecological Implications and Management Strategies: A Review. Sustainability.

[5] Chiutula C., Mtewa A.G., Abraham A., Mvula R.L.S., Maluwa A., Eregno F.E., Njalam’mano J. (2025). Assessment of Heavy Metal Accumulation in Wastewater–Receiving Soil–Exotic and Indigenous Vegetable Systems and Its Potential Health Risks: A Case Study from Blantyre, Malawi. Int. J. Environ. Res. Public Health.

[6] Chames M., Nyutu E.N. (2025). Levels of heavy metals distribution near an urban wastewater in Southeastern Michigan. Sci. Rep..

[7] Hama A.K.H., Mustafa F.S., Omer K.M., Hama S., Hamarawf R.F., Rahman K.O. (2023). Heavy metal pollution in the aquatic environment: Efficient and low-cost removal approaches to eliminate their toxicity: A review. RSC Adv..

[8] Ondrasek G., Shepherd J., Rathod S., Dharavath R., Rashid M.I., Brtnicky M., Shahid M.S., Horvatinec J., Zed R. (2025). Metal contamination–a global environmental issue: Sources, implications & advances in mitigation. RSC Adv..

[9] Harada M. (1995). Minamata disease: Methylmercury poisoning in Japan caused by environmental pollution. Crit. Rev. Toxicol..

[10] Okeke E.S., Enochoghene A., Ezeudoka B.C., Kaka S.D., Chen Y., Mao G., ThankGod Eze C., Feng W., Wu X. (2024). A review of heavy metal risks around e-waste sites and comparable municipal dumpsites in major African cities: Recommendations and future perspectives. Toxicology.

[11] Nuwamanya E., Byamugisha D., Nakiguli C.K., Angiro C., Khanakwa A.V., Omara T., Ocakacon S., Onen P., Omoding D., Opio B. (2024). Exposure and Health Risks Posed by Potentially Toxic Elements in Soils of Metal Fabrication Workshops in Mbarara City, Uganda. J. Xenobiot..

[12] Angiro C., Abila P.P., Omara T. (2020). Effects of industrial effluents on the quality of water in Namanve stream, Kampala Industrial and Business Park, Uganda. BMC Res. Notes..

[13] Ocakacon S., Nyenje P.M., Kalibbala H.M., Kulabako R.N., Nagawa C.B., Omara T., Kyarimpa C., Lugasi S.O., Ssebugere P. (2025). Spatiotemporal Dynamics of Microplastics in Nakivubo Catchment: Implications for the Pollution of Lake Victoria. Microplastics.

[14] Turyagumanawe C. (2022). Assessment of How Establishment of Industries in Kampala City Respects Physical Planning Standards: A Case Study of Nakawa Industrial Area. Ph.D. Thesis.

[15] Deffar S.S., Kumar A., Muliwa A., Pili N.N., Omara T. (2024). Bioethanol production from water hyacinth with isolated thermophilic microbial consortium from Kenya. C. R. Chim..

[16] Huynh A.T., Chen Y.-C., Tran B.N.T. (2021). A Small-Scale Study on Removal of Heavy Metals from Contaminated Water Using Water Hyacinth. Processes.

[17] Durairaj S. (2024). Sorption capacity of *Eichhornia crassipes* (Mart.) Solms for zinc removal from electroplating industry wastewater. Environ. Sci. Pollut. Res..

[18] Tesi G., Ejeromedoghene O., Kpomah B., Ipeaiyeda A. (2024). Sorption of Mn(II) Ions From Wastewater Using Dried and Blended Water Hyacinth (*Eichhornia crassipes*) Roots: Adsorption-Desorption Studies and Kinetics. J. Turk. Chem. Soc. A Chem..

[19] Wang X., Guo X., Li T., Zhu J., Pang J., Xu J., Wang J., Huang X., Gao J., Wang L. (2022). Study on Adsorption Characteristics of Heavy Metal Cd2+ by Biochar Obtained from Water Hyacinth. Pol. J. Environ. Stud..

[20] Sayago U.F.C., Ballesteros V.B., Lozano A.M. (2025). Development of a Treatment System of Water with Cr (VI) Through Models Using E. crassipes Biomass with Iron Chloride. Toxics.

[21] Jahangiri F.M., Moutushi H.T., Moniruzzaman M., Hoque S., Hossain M.E. (2021). Removal of lead from aqueous solutions and wastewaters using water hyacinth (*Eichhornia crassipes*) roots. Water Pract. Technol..

[22] Zhang H., Shen Y., Li M., Zhu G., Feng H., Li J. (2019). Egg shell powders-coated membrane for surfactant-stabilized crude oil-in-water emulsions efficient separation. ACS Sustain. Chem. Eng..

[23] Nimusiima D., Byamugisha D., Omara T., Ntambi E. (2023). Physicochemical and Microbial Quality of Water from the Ugandan Stretch of the Kagera Transboundary River. Limnol. Rev..

[24] Turek A., Wieczorek K., Wolf W.M. (2019). Digestion Procedure and Determination of Heavy Metals in Sewage Sludge—An Analytical Problem. Sustainability.

[25] Zięzio M., Charmas B., Jedynak K., Hawryluk M., Kucio K. (2020). Preparation and characterization of activated carbons obtained from the waste materials impregnated with phosphoric acid (V). Appl. Nanosci..

[26] Danish M., Ahmad T., Nadhari W., Ahmad M., Khanday W.A., Ziyang L., Pin Z. (2018). Optimization of banana trunk-activated carbon production for methylene blue-contaminated water treatment. Appl. Water Sci..

[27] Erlangga R., Sari D.A., Wahyuningtyas A. (2025). Characterization of *Eichhornia crassipes* bio-adsorbent activated by H3PO4 for the removal of lead ion (Pb2+) from wastewater of battery industry. Sinergi.

[28] National Environment Management Authority The National Environment (Standards for Discharge of Effluent into Water or on Land) Regulations 1999. https://nema.go.ug/sites/all/themes/nema/docs/effluent_discharge_regulations.pdf.

[29] Busini V., Florit F., Barozzi M., Sieni E., Copelli S. (2023). Intensification of Processes for the Production of Active Pharmaceutical Ingredients: Increase in Safety and Sustainability. Chem. Eng. Trans..

[30] Jildeh Z.B., Wagner P.H., Schöning M.J. (2021). Sterilization of Objects, Products, and Packaging Surfaces and Their Characterization in Different Fields of Industry: The Status in 2020. Phys. Status Solid..

[31] Kato S., Kansha Y. (2024). Comprehensive review of industrial wastewater treatment techniques. Environ. Sci. Pollut. Res..

[32] Lemessa F., Simane B., Seyoum A., Gebresenbet G. (2023). Assessment of the Impact of Industrial Wastewater on the Water Quality of Rivers around the Bole Lemi Industrial Park (BLIP), Ethiopia. Sustainability.

[33] Walakira P., Okot-Okumu J. (2011). Impact of industrial effluents on water quality of streams in Nakawa-Ntinda, Uganda. J. Appl. Sci. Environ. Manag..

[34] Wanasolo W., Kiremire B.T., Kansiime F. (2018). Evaluation of industrial effluent levels in Kinawataka stream, its tributaries and Kinawataka swamp, prior to discharge into Lake Victoria. Am. J. Chem. Mat. Sci..

[35] Phiri O., Mumba P., Moyo B., Kadewa W. (2005). Assessment of the impact of industrial effluents on water quality of receiving rivers in urban areas of Malawi. Int. J. Environ. Sci. Technol..

[36] Noukeu N.A., Gouado I., Priso R.J., Ndongo D., Taffouo V.D., Dibong S.D., Ekodeck G.E. (2016). Characterization of effluent from food processing industries and stillage treatment trial with *Eichhornia crassipes* (Mart.) and *Panicum maximum* (Jacq.). Water Resour. Ind..

[37] WHO (2022). Guidelines for Drinking-Water Quality: Fourth Edition Incorporating the First and Second Addenda.

[38] Oladimeji T.E., Oyedemi M., Emetere M.E., Agboola O., Adeoye J.B., Odunlami O.A. (2024). Review on the impact of heavy metals from industrial wastewater effluent and removal technologies. Heliyon.

[39] Muwanga A., Barifaijo E. (2010). Impact of industrial activities on heavy metal loading and their physico-chemical effects on wetlands of Lake Victoria basin (Uganda). Afr. J. Sci. Technol..

[40] Chahal C., van den Akker B., Young F., Franco C., Blackbeard J., Monis P. (2016). Pathogen and Particle Associations in Wastewater: Significance and Implications for Treatment and Disinfection Processes. Adv. Appl. Microbiol..

[41] Rajendiran T., Sabarathinam C., Panda B., Elumalai V. (2023). Influence of Dissolved Oxygen, Water Level and Temperature on Dissolved Organic Carbon in Coastal Groundwater. Hydrology.

[42] Tsertou E., Caluwé M., Goettert D., Goossens K., Seguel S.K., Vanherck C., Dries J. (2024). Impact of low and high temperatures on aerobic granular sludge treatment of industrial wastewater. Water Sci. Technol..

[43] Parveen N., Chowdhury S., Goel S. (2022). Environmental impacts of the widespread use of chlorine-based disinfectants during the COVID-19 pandemic. Environ. Sci. Pollut. Res. Int..

[44] Dudziak M., Kudlek E. (2019). Removal of hardness in wastewater effluent using membrane filtration. Archit. Civ. Eng. Envir..

[45] Kinuthia G.K., Ngure V., Beti D., Lugalia R., Wangila A., Kamau L. (2020). Levels of heavy metals in wastewater and soil samples from open drainage channels in Nairobi, Kenya: Community health implication. Sci. Rep..

[46] Khan S.N., Nafees M., Imtiaz M. (2023). Assessment of industrial effluents for heavy metals concentration and evaluation of grass (Phalaris minor) as a pollution indicator. Heliyon.

[47] Mahmood Q., Shaheen S., Bilal M., Tariq M., Zeb B.S., Ullah Z., Ali A. (2019). Chemical pollutants from an industrial estate in Pakistan: A threat to environmental sustainability. Appl. Water Sci..

[48] Government of the Republic of Uganda National Environment (Standards for Discharge of Effluent into Water or Land) Regulations, 2020. Statutory Instruments Supplement No. 4, The Uganda Gazette No. 85, Volume CXIII. Entebbe, Uganda. https://www.nema.go.ug/en/wp-content/uploads/2025/01/National-Environment-Standards-for-Discharge-of-Effluent-into-Water-or-Land-Regulations-2020.pdf.

[49] Torres-Mansilla A., Álvarez-Lloret P., Fernández-Penas R., D’Urso A., Baldión P.A., Oltolina F., Follenzi A., Gómez-Morales J. (2023). Hydrothermal Transformation of Eggshell Calcium Carbonate into Apatite Micro-Nanoparticles: Cytocompatibility and Osteoinductive Properties. Nanomaterials.

[50] Jiang H., Li X., Bai J., Pan W., Luo Z., Dai Y. (2024). Removal of ciprofloxacin lactate by phosphoric acid activated biochar: Urgent consideration of new antibiotics for human health. Chem. Eng. Sci..

[51] Chu G., Zhao J., Huang Y., Zhou D., Liu Y., Wu M., Peng H., Zhao Q., Pan B., Steinberg C.E.W. (2018). Phosphoric acid pretreatment enhances the specific surface areas of biochars by generation of micropores. Environ. Pollut..

[52] Said H.A., Ait Bourhim I., Ouarga A., Iraola-Arregui I., Lahcini M., Barroug A., Noukrati H., Ben Youcef H. (2023). Sustainable phosphorylated microcrystalline cellulose toward enhanced removal performance of methylene blue. Int. J. Biol. Macromol..

[53] Saeb M.R., Ghaffari M., Rastin H., Khonakdar H.A., Simon F., Najafi F., Goodarzi V., Vijayan P.P., Puglia D., Asl F.H. (2017). Biowaste chicken eggshell powder as a potential cure modifier for epoxy/anhydride systems: Competitiveness with terpolymer-modified calcium carbonate at low loading levels. RSC Adv..

[54] Tran H.N., Chao H.-P., You S.-J. (2017). Activated carbons from golden shower upon different chemical activation methods: Synthesis and characterizations. Adsorpt. Sci. Technol..

[55] Wang X.J., Xu X.M., Liang X., Wang Y., Liu M., Wang X., Xia S.Q., Zhao J.F., Yin D.Q., Zhang Y.L. (2011). Adsorption of copper(II) onto sewage sludge-derived materials via microwave irradiation. J. Hazard. Mat..

[56] Currie H.A., Perry C.C. (2007). Silica in plants: Biological, biochemical and chemical studies. Ann. Bot..

[57] Ronsse F., van Hecke S., Dickinson D., Prins W. (2013). Production and characterization of slow pyrolysis biochar: Influence of feedstock type and pyrolysis conditions. Glob. Change Biol. Bioenergy..

[58] Mahato P.L., Weatherby T., Ewell K., Jha R., Mishra B. (2024). Scanning electron microscope-based evaluation of eggshell quality. Poult. Sci..

[59] Nakamoto K. (2008). Applications in Bioinorganic Chemistry. Infrared and Raman Spectra of Inorganic and Coordination Compounds.

[60] Smith B.C. (2018). Infrared Spectral Interpretation: A Systematic Approach.

[61] Noorbakhsh Y., Bozorgghomi S., Ghaemi A. (2025). Simulation and optimization of copper, nickel, cadmium, and zinc removal from industrial wastewater using Aspen adsorption and RSM. Sci. Rep..

[62] Macena M., Pereira H., Cruz-Lopes L., Grosche L., Esteves B. (2025). Competitive Adsorption of Metal Ions by Lignocellulosic Materials: A Review of Applications, Mechanisms and Influencing Factors. Separations.

[63] Gkika D.A., Toubanaki D.K., Thysiadou A.A., Kyzas G.Z., Tolkou A.K. (2026). Toward Circular and Sustainable Urban Wastewater Treatment: Integrating Adsorption and Advanced Oxidation Processes. Urban Sci..

[64] Zhuo G.H., Xue D.X., Huang L.F., Wang Q., Zhu G., Wang C.Y. (2025). Adsorption of phosphates on a novel eggshell Ca-modified anaerobic sludge-based biochar: Adsorption performance and mechanism. PLoS ONE.

[65] Kuśmierek K., Dąbek L., Świątkowski A. (2025). The influence of the shape and grain size of commercial activated carbons on their sorption efficiency towards organic water pollutants. Desalin. Water Treat..

[66] Gheibi M., Masoomi S.R., Eftekhari M., Akrami M., Palušák M., Silvestri D., Černík M., Wacławek S. (2025). Heavy metals adsorption using CDW adsorbents: A sustainable path for water purification. J. Hazard. Mat. Adv..

[67] Khan M., Shafi M., Raza J., Ahmed I.A., Zada A., Narasimharao K., Sun X. (2025). Mechanistic breakthroughs in affordable adsorbents for heavy metal remediation: An in-depth exploration of next-generation sustainable water purification technologies. J. Hazard. Mat. Adv..

[68] Wu Q., Miao X., Zhu Z. (2025). Investigation into the adsorption performance of tobermorite with varying calcium-silicon ratios for Cr^3+^ and Cu^2+^ ions. Desalin. Water Treat..

[69] Weyrich J.N., Mason J.R., Bazilevskaya E.A., Yang H. (2023). Understanding the Mechanism for Adsorption of Pb(II) Ions by Cu-BTC Metal-Organic Frameworks. Molecules.

[70] Abdelrhman F., Ram S., Zhou J., Ahmed Z., Altaf N.-U., Mostafa E., Zhang Y. (2026). Chemically modified biochar for enhanced heavy metals adsorption in aqueous solutions. Resour. Chem. Mater..

[71] Musumba G., Nakiguli C., Lubanga C., Mukasa P., Ntambi E. (2020). Adsorption of Lead (II) and Copper (II) Ions from Mono Synthetic Aqueous Solutions Using Bio-Char from Ficus natalensis Fruits. J. Encapsul. Adsorpt. Sci..

[72] Sabone B., Letshwenyo M.W., Mokgosi S. (2026). Investigation of plant seeds as adsorbents for lead and copper ions from aqueous solutions: Batch studies. Sep. Sci. Technol..

[73] Afkhami A., Saber-Tehrani M., Bagheri H. (2010). Simultaneous removal of heavy-metal ions in wastewater samples using nano-alumina modified with 2,4-dinitrophenylhydrazine. J. Hazard. Mat..

