# Supplementary material for: Eggshell-Activated Carbon from Water Hyacinths for Heavy Metal Removal from Wastewater: Isotherm and Kinetic Studies
Source: J Xenobiot. 2026 Jul 8;16(4):126. doi: 10.3390/jox16040126 (PMC13397911; doi:10.3390/jox16040126)
Supplement: Supplementary file 1 [file jox-16-00126-s001.zip › jox-4364633-supplementary.pdf]

Supplementary Materials: Eggshell-activated Carbon From Water Hyacinths for Heavy Metal Removal from Wastewater: Isotherm and Kinetic studies

Claire Atumanye [1], Simon Bbumba [2,3], Hakimu Nsubuga [4], Ivan Kiganda [2,*], Timothy Omara [2,*] and Justus Kwetegyeka [1,*]

Text S1: Batch adsorption studies

S1.1. Effect of adsorbent type

A measured volume of 100 ml of each prepared working solution was placed in separate 500 ml beakers. Then, 0.5 g of EP–WH–PH (300 μm) powder was added, the mixture was sonicated for 5 minutes and allowed an adsorption time of 2 hours. The mixtures were filtered and the concentration of the HMs in the filtrates determined using AAS. The obtained concentrations were used to determine the absorption efficiencies of the EP–WH–PA, EP–WH and WHB adsorbents for each of the HMs. The effect of adsorbent type was further assessed based on the surface area, and functional groups based on the results obtained from SEM-EDX. The adsorbent with the best adsorption physicochemical characteristics and the highest adsorption efficiency (q; equations 1 and 2 [19]) was chosen for further experiments.

$$q = \frac{C_0 - C_1}{C_0} \times 100 \quad (1)$$

$$q_e = \frac{(C_0 - C_1)V}{W} \quad (2)$$

where C_0 = initial heavy metal concentration, C_1 = final heavy metal concentration in WW, V = volume of aqueous solution (L), and W is the mass of the adsorbent (g).

S1.2. Effect of particle size

In 500 ml beakers, 0.5 g of activated 300 μm WH was added. Then, 100 ml of metal ions from working solutions were added to each beaker. The corresponding mixtures were sonicated for 5 minutes and left to stand for 2 hours to enable adsorption to take place. The mixtures were then filtered. The concentrations (C_2) of HMs in the filtrate were determined using AAS. The procedure was repeated using ground WH with a particle size of 425 μm. The ratio of the amount of adsorbent required for adsorption expressed in milligrams to the amount of adsorbate in mg was used to calculate the efficiency of adsorption (q_e). The effect of pH, contact time, and adsorbent dosage employed the particle size that corresponded to the maximum adsorption efficiency.

S1.3. Effect of solution pH

To five 500 ml beakers each containing 0.5 g of activated WH with an average particle size of 300 μm, 100 ml of metal ion solutions were added. Either 0.1M NaOH or 0.1M H_2SO_4 was used to adjust the pH of the mixture, and a benchtop OHAUS Aqua searcher pH meter was used to measure the pH. The pH values were set at 3, 4, 5, 6 and 7, and the mixtures were sonicated for 5 minutes before being let to stand for 2 hours to enable adsorption to take place. The mixture was filtered and AAS was used to determine the concentrations of the heavy metal ions in the filtrate.

S1.4. Effect of contact time

In ten 500 mL beakers, 0.5 g of the adsorbent was added. A measured volume of 100 mL of the metal ions, with a pH set at the optimum values obtained from the results for the effect of pH (4 for Pb, Cr, and Cu and 5 for Zn and Cd) were added to each beaker. Adsorption was given a set amount of time (10, 20, 30, 40, 50, 60, 70, 80, 90, 100, 110 and 120) minutes. Afterwards, the mixtures were filtered, and the concentrations of the heavy metal ions in the filtrate were measured.

S1.5. Effect of adsorbent dosage

In ten 500 ml beakers, 0.25 g of the adsorbents activated with 300 μm WH powder was added. A measured volume of 100 ml of HM solution was added to each beaker. The mixtures were allowed a contact time of 20 minutes for lead, 40 minutes for Cr, 90 minutes for Cu, and 30 minutes for both Zn and Cd and then filtered. The concentrations of the HMs were determined. With 0.75, 0.5, 1.0, 1.25, 1.75 and 2.0 g of the adsorbents, the above process was repeated.

Table S1. Calibration curve parameters for the analysis of heavy metals in the samples.

Metal	Calibration function	Coefficient of determination (R^2)
Cd	$y = 0.0095x + 0.028$	0.997
Cr	$y = 0.0085x + 0.0006$	0.9972
Cu	$y = 0.009x + 0.0260$	0.9947
Pb	$y = 0.0096x + 0.0176$	0.9981
Zn	$y = 0.004x + 0.0952$	0.9989

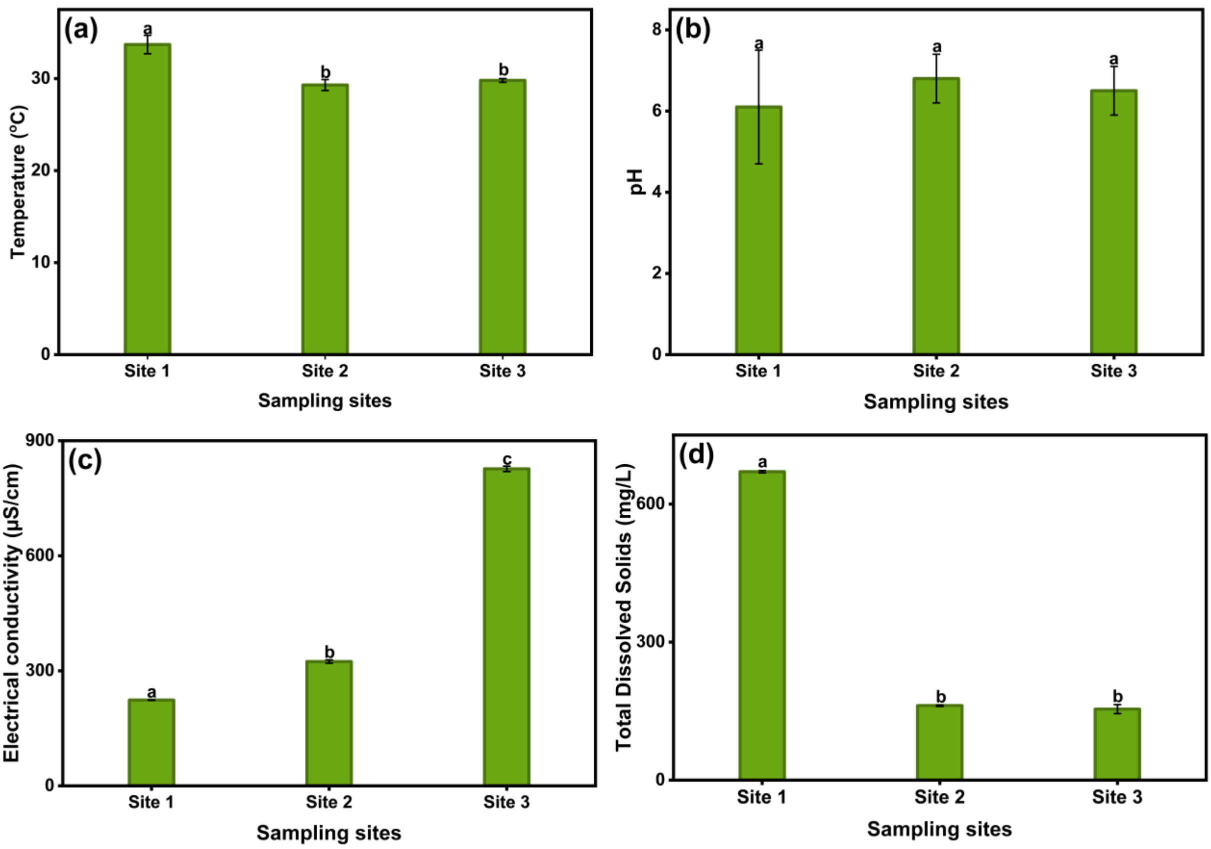

Figure S1. Non-conservable physicochemical parameters of the wastewater samples' (a) temperature, (b) pH, (c) electrical conductivity, and (d) total dissolved solids. For each parameter, bars carrying different alphabetical letters are statistically different ($P<0.05$).

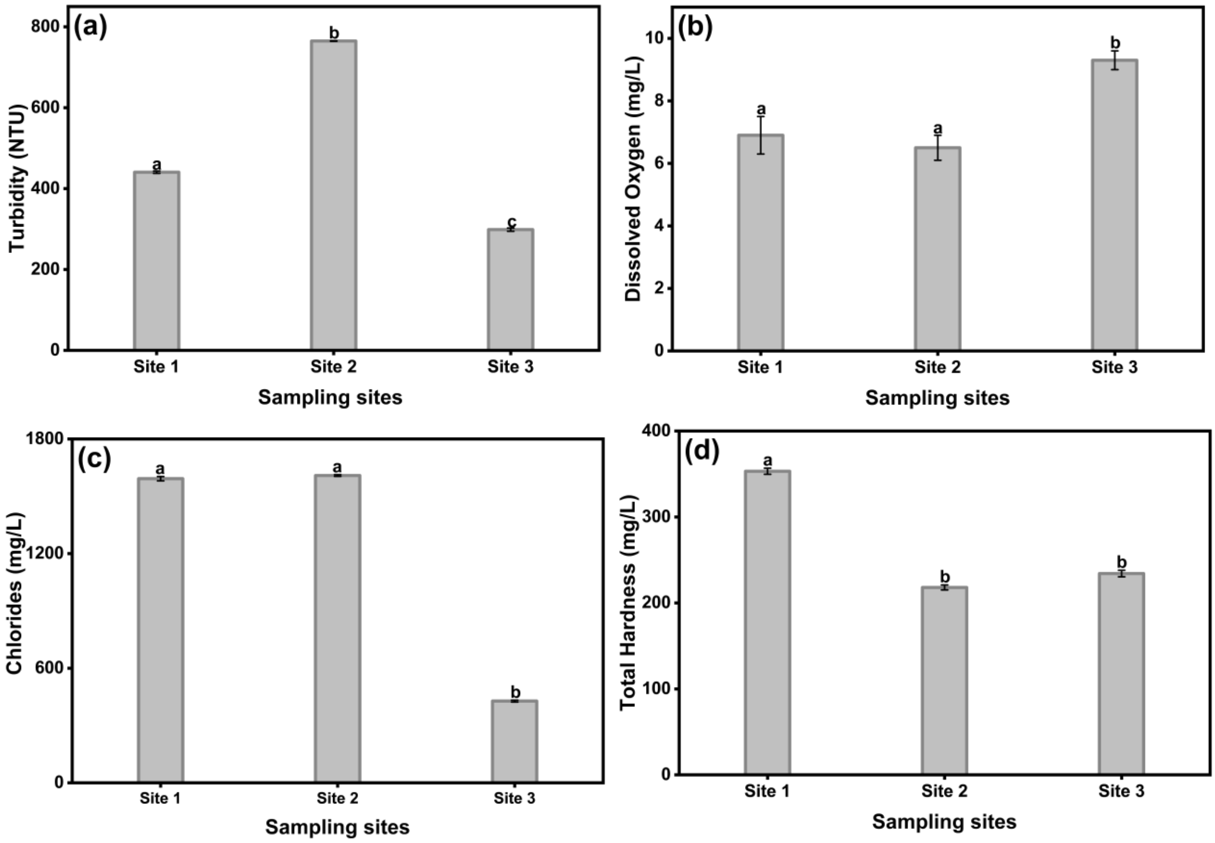

Figure S2. Conservable chemical parameters of the wastewater samples' (a) turbidity, (b) dissolved oxygen, (c) chlorides, and (d) total hardness. For each parameter, bars carrying different alphabetical letters are statistically different ($P<0.05$).

Table S2. Elemental composition of the water hyacinth-based adsorbents.

Water hyacinth biochar	Element	Line	Weight (%)	Atomic (%)	Error (%)	Net Intensity	R	A	F
Unactivated	C K	K	92.82	94.56	7.39	111.59	0.9476	0.5985	1.0000
	O K	K	7.04	5.38	24.44	7.28	0.9556	0.2298	1.0000
	Si K	K	0.14	0.06	76.63	0.51	0.9724	0.9278	1.0055
Eggshell-treated activated	C K	K	70.59	78.10	8.62	61.78	0.9395	0.5241	1.0000
	O K	K	24.21	20.11	13.61	23.85	0.9486	0.2630	1.0000
	Si K	K	0.48	0.23	44.52	1.43	0.9675	0.9088	1.0076
	Ca K	K	4.72	1.56	19.69	4.17	0.9819	0.9916	1.0243
Double activated (EP–WH–PA)	C K	K	12.86	21.96	16.57	3.78	0.8965	0.2599	1.0000
	O K	K	40.93	52.45	11.16	26.85	0.9099	0.2578	1.0000
	Si K	K	0.51	0.37	32.38	1.01	0.9391	0.8673	1.0206
	P K	K	12.17	8.06	5.69	17.78	0.9433	0.9093	1.0174
	Ca K	K	33.53	17.15	5.72	19.72	0.9632	0.9766	1.0108

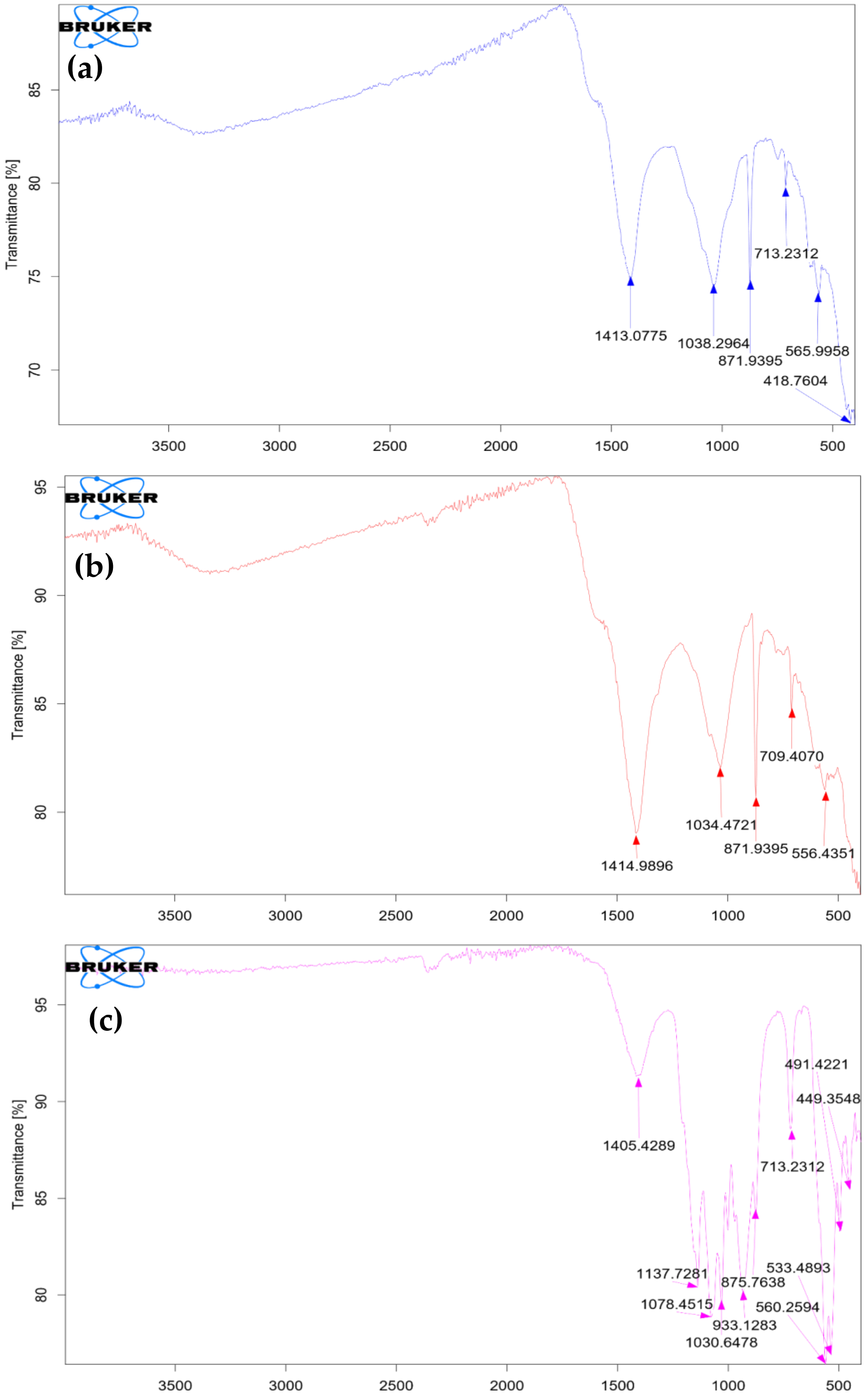

Figure S3. FTIR spectrum of (a) unactivated water hyacinth biochar (WHB), (b) eggshell-treated activated water hyacinth biochar (EP–WH), and (c) double-activated water hyacinth biochar (EP–WH–PA).

Table S3. Effect of adsorbent type on the adsorption of the HMs.

Site	Metal	Heavy metal concentration (mg L^{-1})				Adsorption efficiency (%)		
		C_0	C_1 (WHB)	C_1 (EP-WH)	C_1 (EP-WH-PA)	WHB	EP–WH	EP–WH–PA
1	Cd	7.54	1.22	0.96	0.24	83.8	87.3	96.8
	Cr	0.54	<LOD	<LOD	<LOD	100	100	100
	Pb	4.75	<LOD	<LOD	<LOD	100	100	100
	Zn	0.91	<LOD	<LOD	<LOD	100	100	100
	Cu	0.64	<LOD	<LOD	<LOD	100	100	100
2	Cd	40.18	10.47	8.03	6.58	73.9	80.0	83.6
	Cr	8.21	1.47	0.72	0.49	82.1	91.2	94.0
	Pb	72.03	23.40	19.02	17.03	67.5	73.6	76.4
	Zn	63.01	20.02	18.92	14.45	68.2	69.97	77.1
	Cu	8.73	1.30	1.08	0.86	85.1	87.6	90.2
3	Cd	77.13	25.64	20.92	18.24	66.8	72.9	76.4
	Cr	13.07	1.97	1.16	0.93	84.9	91.1	92.9
	Pb	93.54	38.02	36.94	32.84	59.4	60.5	64.9
	Zn	84.62	27.98	26.77	24.12	66.9	68.4	71.5
	Cu	54.03	14.62	11.27	10.07	72.9	79.1	81.4

Note: LOD = Limit of detection.

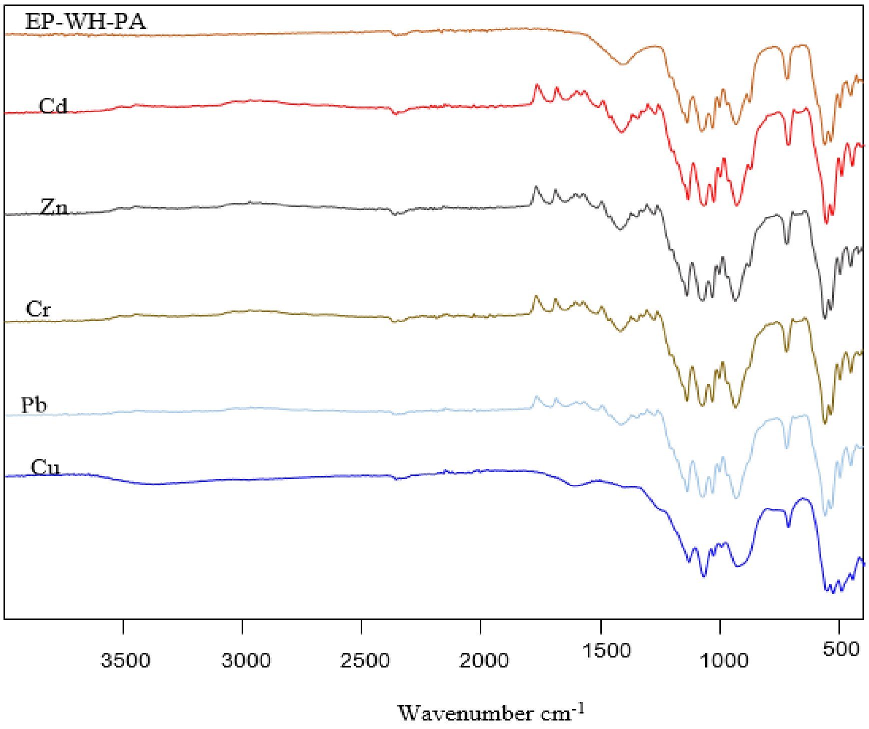

Figure S4. FTIR spectra after adsorption of selected metal ions by the activated water hyacinth biochar (EP–WH–PA).

Table S4. Effect of adsorbent particle size on the adsorption of HMs.

Metal	Concentration (mg L^{-1})	Particle sizes	
		300 μm	425 μm
		Adsorption efficiency (%)	
	7.54	96.8	94.3
Cd	40.18	83.6	78.4
	77.13	76.4	66.4
	4.75	100	96.4
Pb	72.03	76.4	70.5
	93.54	64.9	51.9
	0.91	100	100
Zn	63.01	77.1	67.9
	84.62	71.5	58.0
	0.64	100	100
Cu	8.73	90.1	85.6
	54.03	81.4	72.8
	0.54	100	100
Cr	8.21	94.0	93.2
	13.07	92.9	90.2

Table S5. Effect of solution pH on the adsorption of HMs.

Metal	Concentration (mg L^{-1})	Solution pH				
		3	4	5	6	7
		Adsorption efficiency (%)				
	7.54	95.8	96.3	96.3	96.8	96.8
Cd	40.18	61.7	68.6	83.6	83.7	83.6
	77.13	47.4	56.2	75.9	76.5	76.4
	4.75	100	100	100	100	100
Pb	72.03	63.8	76.4	76.4	76.4	73.7
	93.54	51.8	64.9	64.9	64.9	62.9
	0.91	100	100	100	100	100
Zn	63.01	51.4	60.9	77.1	77.1	77.1
	84.62	44.8	55.1	71.5	71.5	71.5
	0.64	100	100	100	100	100
Cu	8.73	89.2	90.1	90.1	90.1	88.2
	54.03	74.6	81.4	81.4	81.4	77.3
Cr	0.54	100	100	100	100	100
	8.21	93.1	94	94	93.9	93.7

13.07	91.6	92.9	92.9	92.9	92.7

Table S6. Effect of contact time on the adsorption of HMs.

Metal	Concentration (mg L^{-1})	Time (minutes)											
		10	20	30	40	50	60	70	80	90	100	110	120
		Adsorption efficiency (%)											
Cd	7.54	95.1	96.2	96.8	96.8	96.8	96.8	96.8	96.8	96.8	96.8	96.8	96.8
	40.18	53.6	73.9	83.7	83.7	83.7	83.7	83.7	83.7	83.7	83.7	83.7	83.7
	77.13	40.7	66.8	76.4	76.4	76.4	76.4	76.4	76.4	76.4	76.4	76.4	76.4
Pb	4.75	100	100	100	100	100	100	100	100	100	100	100	100
	72.03	76.4	76.4	76.4	76.4	76.4	76.4	76.4	76.4	76.4	76.4	76.4	76.4
	93.54	57.7	64.9	64.9	64.9	64.9	64.9	64.9	64.9	64.9	64.9	64.9	64.9
Zn	0.91	100	100	100	100	100	100	100	100	100	100	100	100
	63.01	71	74.5	77.1	77.1	77.1	77.1	77.1	77.1	77.1	77.1	77.1	77.1
	84.62	61.1	67.3	69.54	71.5	71.5	71.5	71.5	71.5	71.5	71.5	71.5	71.5
Cu	0.64	100	100	100	100	100	100	100	100	100	100	100	100
	8.73	90.1	90.1	90.3	90.1	90.1	90.1	90.1	90.1	90.1	90.1	90.1	90.1
	54.03	71.9	81.4	81.4	81.4	81.4	81.4	81.4	81.4	81.4	81.4	81.4	81.4
Cr	0.54	100	100	100	100	100	100	100	100	100	100	100	100
	8.21	89	94	94	94	94	94	94	94	94	94	94	94
	13.07	80.1	88.4	90.3	92.9	92.9	92.9	92.9	92.9	92.9	92.9	92.9	92.9

Table S7. Effect of adsorbent dosage on the adsorption of HMs.

Metal	Concentration (mg/L)	Adsorbent dosage (g)							
		0.25	0.5	0.75	1.0	1.25	1.5	1.75	2.0
		Adsorption efficiency (%)							
Cd	7.54	84.5	96.8	100	**100**	100	100	100	100
	40.18	56.2	83.7	95.4	**95.4**	95.4	95.4	95.4	95.4
	77.13	43.4	76.4	85	**90.2**	90.2	90.2	90.2	90.2
Pb	4.75	100	100	100	**100**	100	100	100	100
	72.03	69.4	76.4	82.4	**87.4**	87.4	87.4	87.4	87.4
	93.54	49.6	64.9	67.8	**71.3**	76.8	76.8	76.8	76.8
Zn	0.91	100	100	100	**100**	100	100	100	100
	63.01	70.3	77.1	85.6	**92.2**	92.2	92.2	92.2	92.2
	84.62	65.1	71.5	78.3	**83.4**	83.4	83.4	83.4	83.4
Cu	0.64	100	100	100	**100**	100	100	100	100
	8.73	81.6	90.5	100	**100**	100	100	100	100
	54.03	72.1	81.5	87.1	**91.8**	91.8	91.8	91.8	91.8
Cr	0.54	100	100	100	**100**	100	100	100	100
	8.21	85.4	94	100	**100**	100	100	100	100
	13.07	80	92.9	900	**100**	100	100	100	100

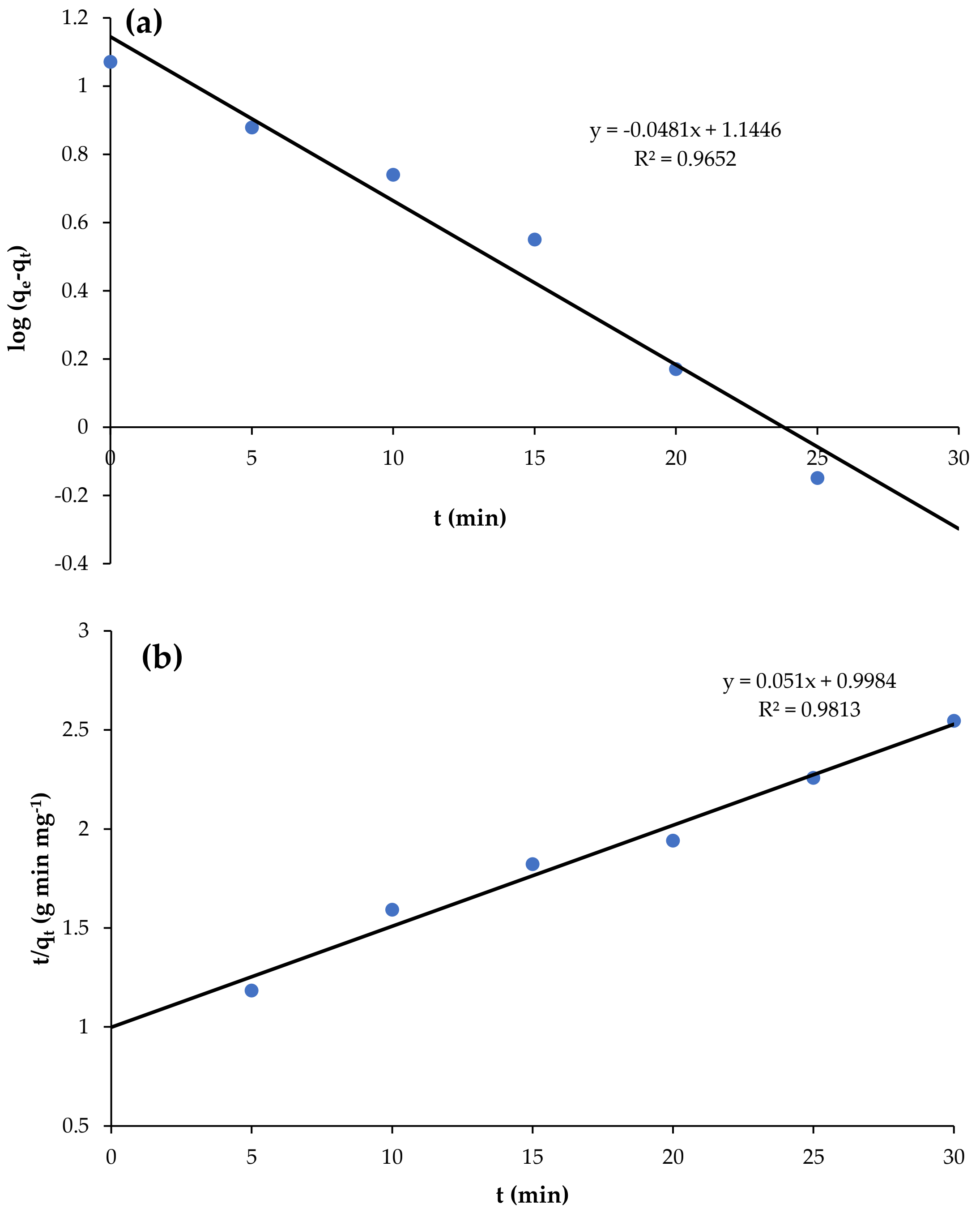

Figure S5. Linearized (a) pseudo-first order, and (b) pseudo-second order kinetic plots for the adsorption of Cd^{2+} ions (77.13 mg L^{-1}) onto eggshell-activated carbon derived from water hyacinths. .

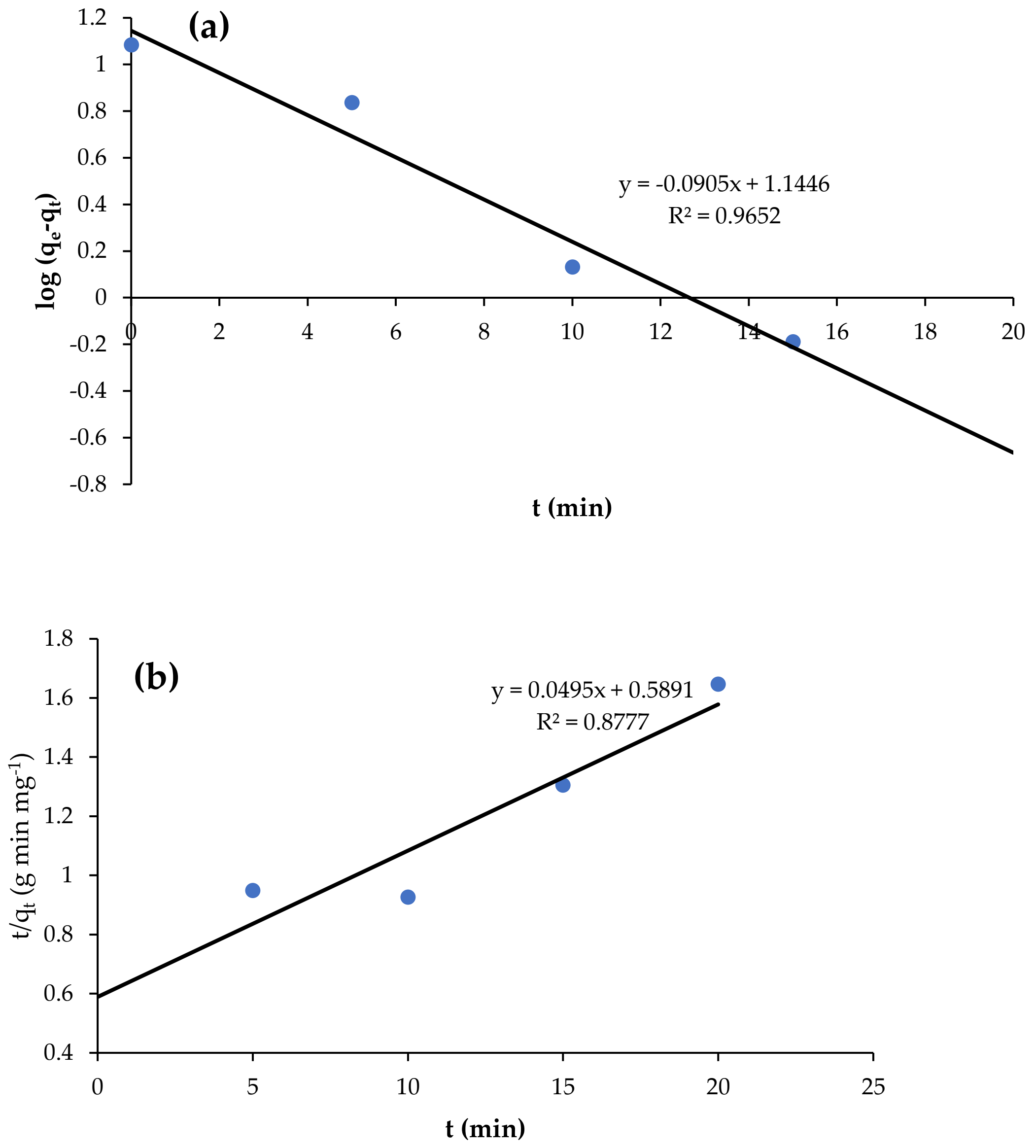

Figure S6. Linearized (a) pseudo-first order, and (b) pseudo-second order kinetic plots for the adsorption of Pb^{2+} ions (93.54 mg L^{-1}) onto eggshell-activated carbon derived from water hyacinths. .

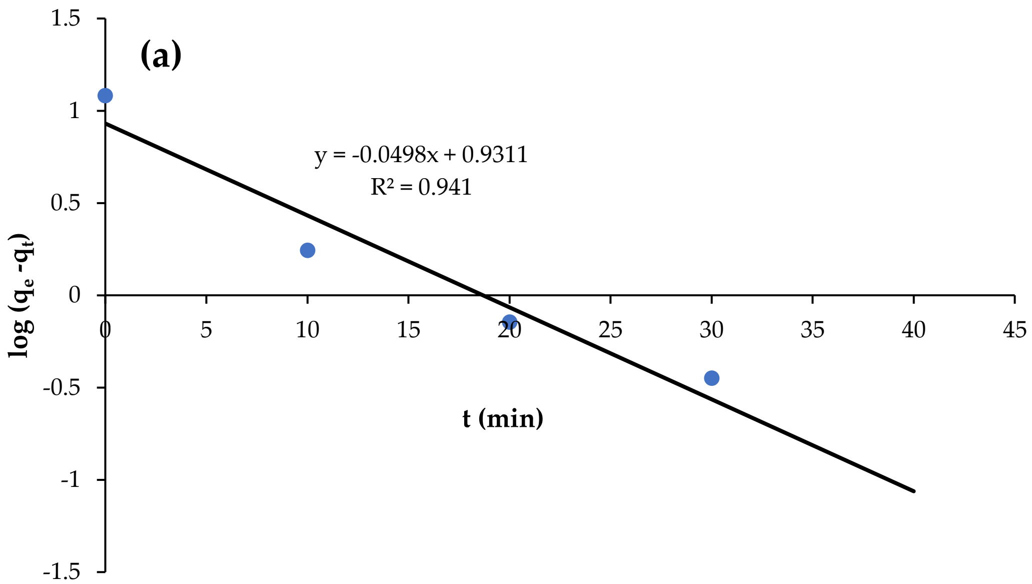

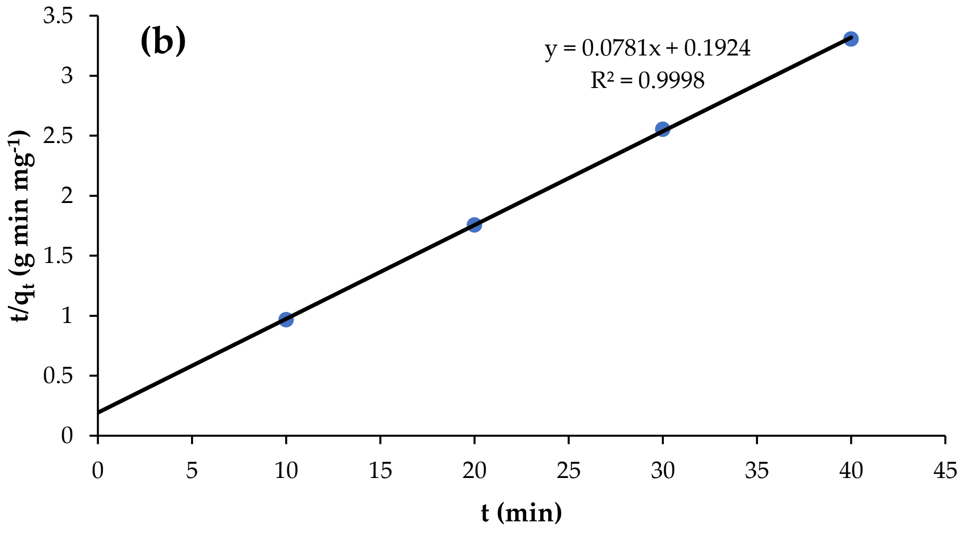

Figure S7. Linearized (a) pseudo-first order, and (b) pseudo-second order kinetic plots for the adsorption of Zn^{2+} ions (63.01 mg L^{-1}) onto eggshell-activated carbon derived from water hyacinths.

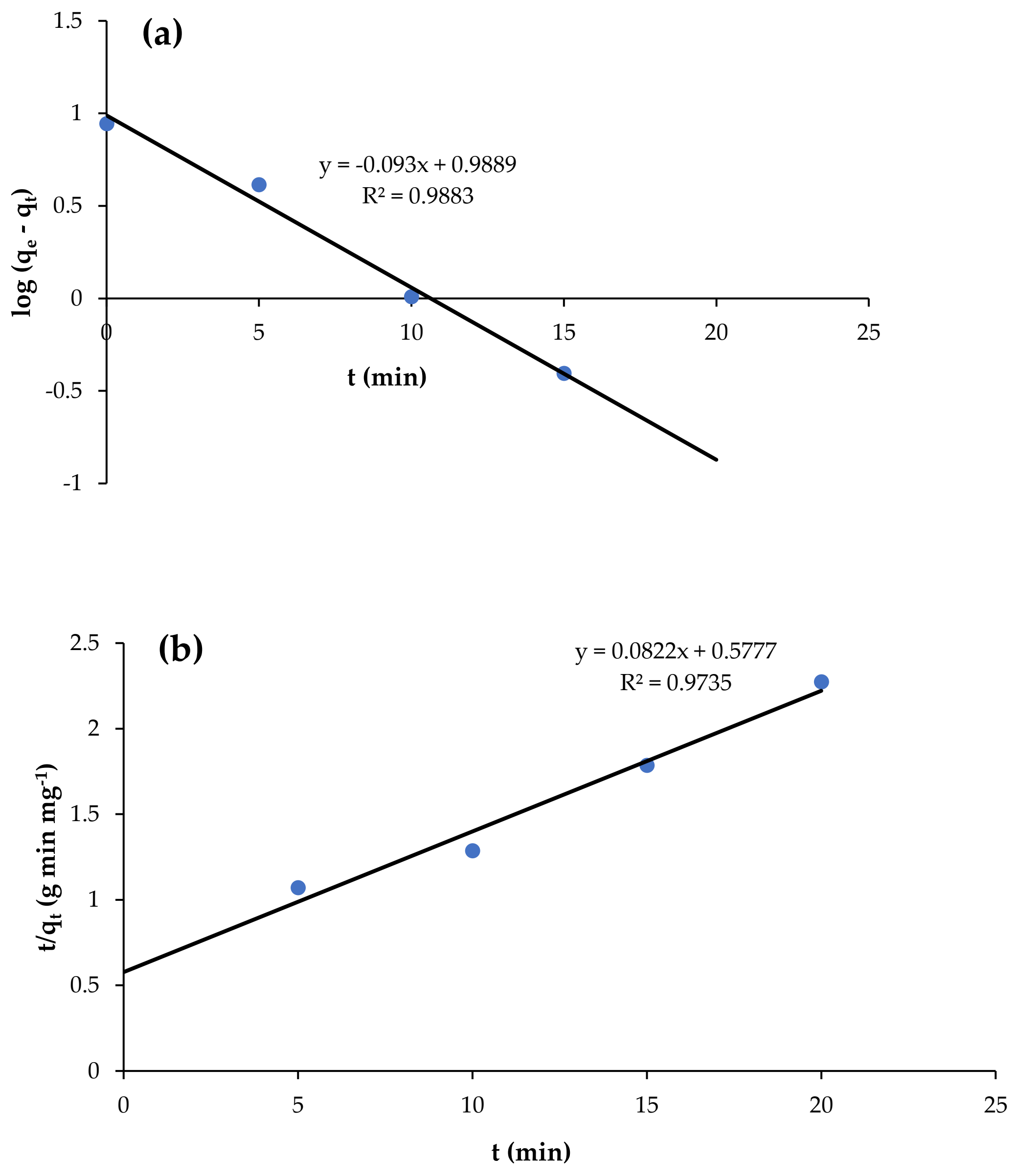

Figure S8. Linearized (a) pseudo-first order, and (b) pseudo-second order kinetic plots for the adsorption of Cu^{2+} ions (54.03 mg L^{-1}) onto eggshell-activated carbon derived from water hyacinths.

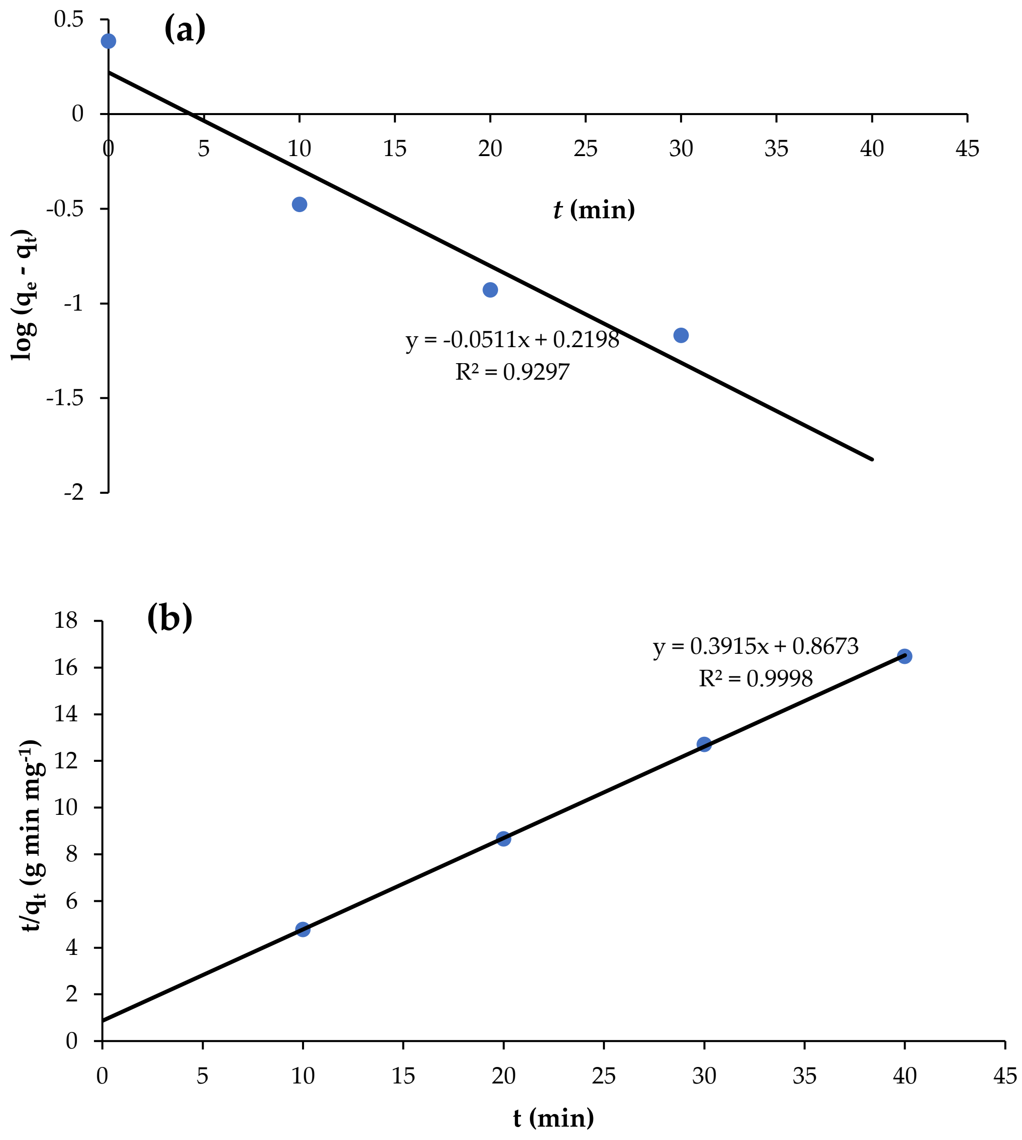

Figure S9. Linearized (a) pseudo-first order, and (b) pseudo-second order kinetic plots for the adsorption of Cr^{3+} ions (13.07 mg L^{-1}) onto eggshell-activated carbon derived from water hyacinths.

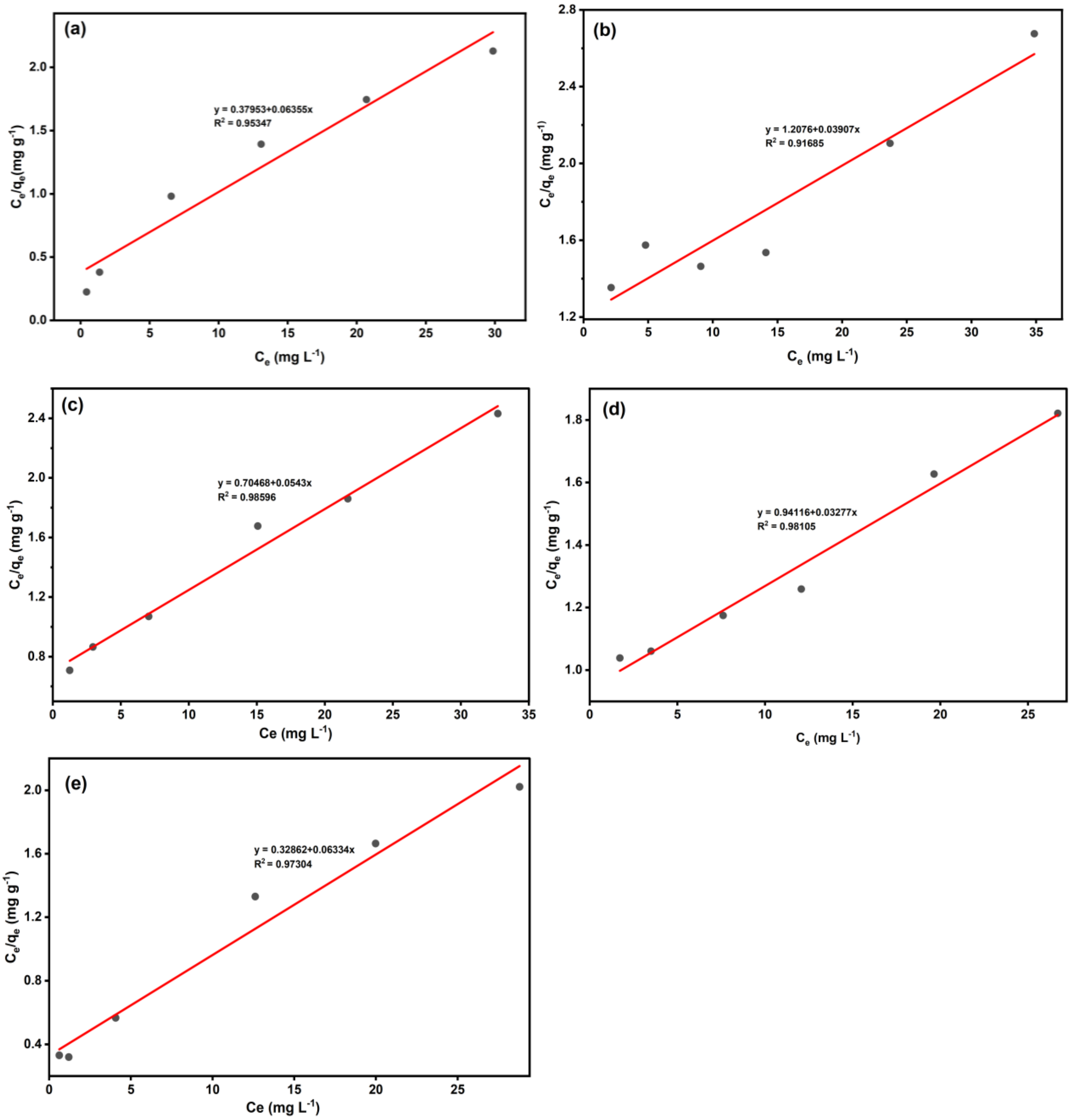

Figure S10. Linearized Langmuir isotherm plots for the adsorption of (a) Cd^{2+}, (b) Pb^{2+}, (c) Zn^{2+}, (d) Cu^{2+}, and (e) Cr^{3+} ions onto eggshell-activated carbon derived from water hyacinths.

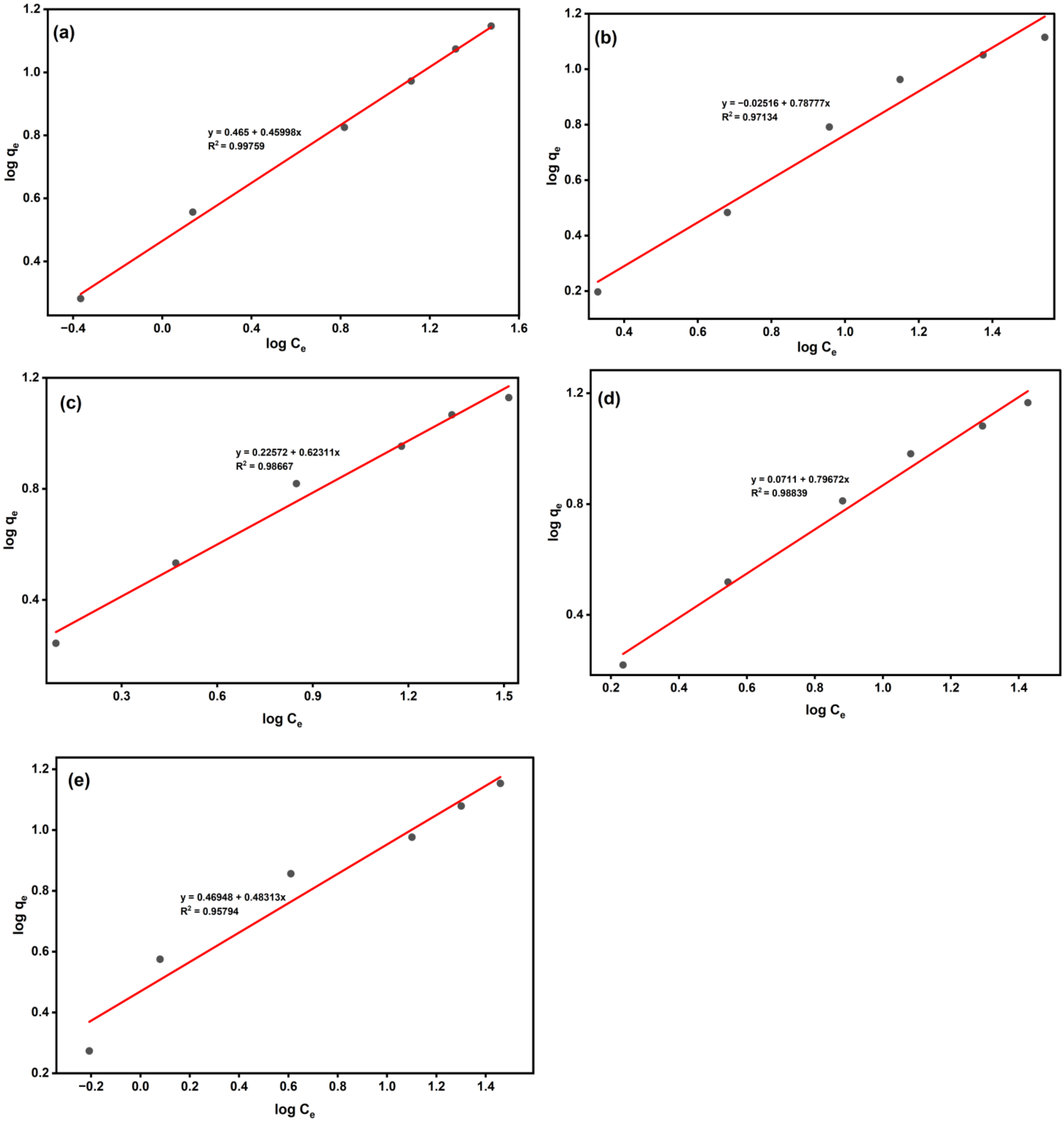

Figure S11. Linearized Freundlich isotherm plots for the adsorption of (a) Cd^{2+}, (b) Pb^{2+}, (c) Zn^{2+}, (d) Cu^{2+}, and (e) Cr^{3+} ions onto eggshell-activated carbon derived from water hyacinths.

References

19 Wang, X.; Guo, X.; Li, T.; Zhu, J.; Pang, J.; Xu, J.; Wang, J.; Huang, X.; Gao, J.; Wang, L. Study on Adsorption Characteristics of Heavy Metal Cd2+ by Biochar Obtained from Water Hyacinth. *Pol. J. Environ. Stud.* **2022**, *31*, 2301–2316.
